# Prediction of Biofilm Inhibiting Peptides: An *In silico* Approach

**DOI:** 10.3389/fmicb.2016.00949

**Published:** 2016-06-16

**Authors:** Sudheer Gupta, Ashok K. Sharma, Shubham K. Jaiswal, Vineet K. Sharma

**Affiliations:** Metagenomics and Systems Biology Group, Department of Biological Sciences, Indian Institute of Science Education and Research BhopalBhopal, India

**Keywords:** biofilm, support vector machine, machine learning, peptides, random forest

## Abstract

Approximately 75% of microbial infections found in humans are caused by microbial biofilms. These biofilms are resistant to host immune system and most of the currently available antibiotics. Small peptides are extensively studied for their role as anti-microbial peptides, however, only a limited studies have shown their potential as inhibitors of biofilm. Therefore, to develop a unique computational method aimed at the prediction of biofilm inhibiting peptides, the experimentally validated biofilm inhibiting peptides sequences were used to extract sequence based features and to identify unique sequence motifs. Biofilm inhibiting peptides were observed to be abundant in positively charged and aromatic amino acids, and also showed selective abundance of some dipeptides and sequence motifs. These individual sequence based features were utilized to construct Support Vector Machine-based prediction models and additionally by including sequence motifs information, the hybrid models were constructed. Using 10-fold cross validation, the hybrid model displayed the accuracy and Matthews Correlation Coefficient (MCC) of 97.83% and 0.87, respectively. On the validation dataset, the hybrid model showed the accuracy and MCC value of 97.19% and 0.84, respectively. The validated model and other tools developed for the prediction of biofilm inhibiting peptides are available freely as web server at http://metagenomics.iiserb.ac.in/biofin/ and http://metabiosys.iiserb.ac.in/biofin/.

## Introduction

Biofilms are surface associated well-structured multicellular communities of microorganisms (e.g., archaea, bacteria, fungi, and algae), capable of growing on diverse range of biotic and abiotic surfaces, and encased in self-secreted extra cellular matrix called extra polymeric substance (EPS; Hall-Stoodley et al., 2004). Presently, they are one of the major cause of health problems worldwide because of two main reasons, firstly: ~75% of all human infections (majorly chronic) are caused by these biofilms, and secondly: due to the multicellular, robust and protected structure, they are resistant (up to 100-fold more than planktonic bacteria) to host defense mechanisms and traditional antimicrobials which largely targets planktonic bacteria (Costerton et al., 1999; Mah and O'Toole, 2001; Davies, 2003; de la Fuente-Nunez et al., 2013).

Formation of biofilm is primarily dependent on the ability of microbes to communicate and co-operate with other cells via quorum sensing, which is done by releasing and responding to small diffusible signal molecules (Li and Tian, 2012). After dramatic success of antimicrobial peptides (AMP) as anti-microbial agents against free-swimming bacteria, the interest in considering AMPs for the treatment of biofilm is increasing, for example, *Staphylococcus aureus* and *Staphylococcus epidermidis* associated biofilm is effectively inhibited by Ribonucleic-acid-III-inhibiting peptide (Balaban et al., 2005) and human cathelicidin peptide (Mishra et al., 2016). More precisely, the biofilm inhibiting peptides (BIPs) are a class of AMPs which can independently inhibit multiple steps, including quorum sensing, inhibition of cell adhesion to the other cells and surfaces, activation of genes responsible for motility, down-regulation of genes responsible for production of EPS and causing direct bacterial killing (Ding et al., 2014; Brackman and Coenye, 2015; Wu et al., 2015). Additionally, ability of BIPs to target specific physiological features of biofilm forming cells and specific stages of biofilm formation underscores their significance (de la Fuente-Nunez et al., 2012). BIPs can target plasma membrane as well as the intracellular targets, for example, magainin, buforin II, and pleurocidin can target cell membrane lipopolysaccharides as well as the intracellular DNA (Vorland et al., 1999; Lan et al., 2010).

Many of the BIPs have already been tested as prophylactic and therapeutic agents against the biofilms both *in vitro* and *in vivo* (Batoni et al., 2011; Dosler and Karaaslan, 2014; de la Fuente-Nunez et al., 2015). They are attractive therapeutic agents because of their ability to act rapidly on a broad range of bacteria, including slow-growing and non-growing bacteria (Dosler et al., 2016). Furthermore, due to their multifaceted action on common and conserved pathways, the frequency of selection of resistant strains toward BIPs is slow (Batoni et al., 2011). Several naturally occurring BIPs have been reported from a diverse range of organisms, such as humans—HBD3, AMP-IBP5, LL-37, and α-MSH, other mammals—cathelicidin WAM1BM and AP-28, arthropods—tachyplesin III, amphibians—magainin I, aurein 2.5 and phylloseptin-1, fish—pleurocidin and chrysophsin-1, bacteria—lacticin 3147, gramicidin A and nisin, and plants—*Tn*-AFP1 (Jorge et al., 2012; de la Fuente-Nunez et al., 2016). Furthermore, multiple synthetic BIPs have also been reported, such as synthetic—F2,5,12W, KSL, Tet213, PTP-7, SAMPs Ltx5, Ltx9, and Ltx10, mimetics—peptoid 1-C134mer, peptoid 1, and (RW)4D, omiganan pentahydrochloride—STAMPs C16G2, M8-33, M8G2, C16-33, and G10KHc (Jorge et al., 2012).

The current focus is mainly toward synthetic BIPs, optimizing their performance and designing more potent biofilm inhibitory peptides. Thus, new computational tools as well as experimental techniques are needed for the identification of novel BIPs which could be used as effective therapeutic agents. In this scenario, a high throughput, robust, cost effective, and efficient tool is desired for the identification of novel and effective BIPs. The prime focus of the available computational tools is on the prediction of AMPs, and no tool is available which is specific for the prediction of BIPs. However, a database of biofilm inhibitory peptides known as BaAMPs (http://www.baamps.it/) is available (Di Luca et al., 2015). In this study, we have exploited the sequence features of all the available experimentally validated BIPs sequences from BaAMPs, and used these features to develop machine learning based prediction models using different approaches, such as Support Vector Machine (SVM) and Random Forest (RF). Based on the evaluation of models using 10-fold cross validation and performance evaluation on validation dataset, the most accurate model was selected to create the web server based tool for the prediction of BIPs.

## Materials and methods

### Preparation of dataset

**Figure 1:**
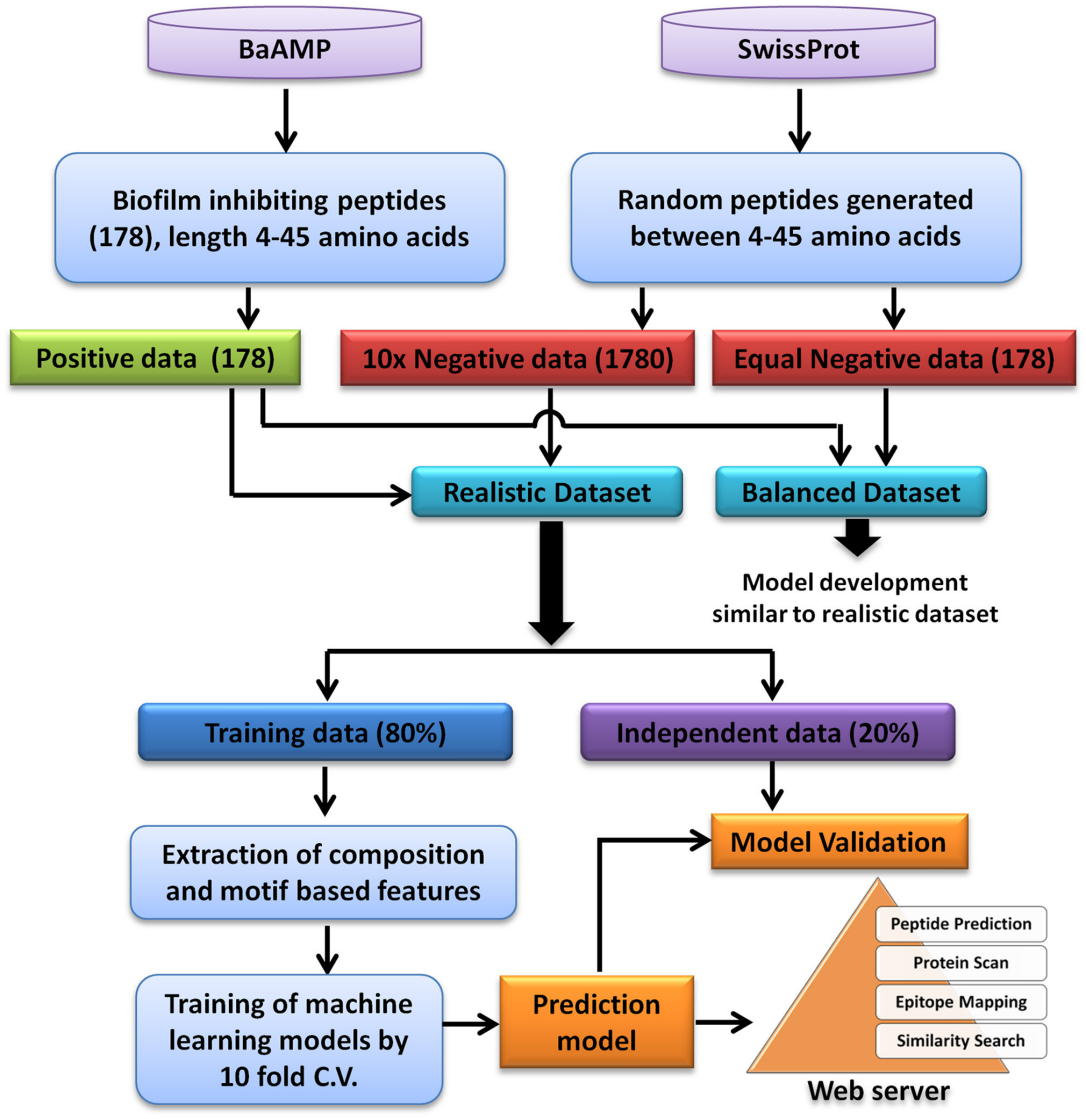
**Flowchart showing steps involved in the development of prediction model and web server**.

A total of 179 unique peptides were downloaded from BaAMP database, which is a comprehensive database of BIPs and their assays. In order to analyse and predict BIP, a length range of 4–45 amino acids was selected since almost all the sequences were lying in this length range except one peptide which was 53 amino acids long. Using this amino acids length, 178 unique biofilm inhibiting peptides were identified. These 178 biofilm inhibiting peptides belonged to 84 different species/strains of biofilm forming bacteria and were considered as the “positive dataset” (Supplementary Table S4). In absence of experimentally validated biofilm non-inhibiting peptides, a set of peptide sequences of 4–45 amino acid length were randomly generated from all SwissProt database sequences. In order to generate the “negative dataset” from random peptide sequences, those sequences which were either exact match or contained the positive peptides were removed from random peptides dataset. To consider the chances of skewness of the prediction models as well as the realistic prediction condition, two types of dataset were generated: (1) Balanced dataset: equal number of positive and negative instances, and (2) Realistic dataset: negative instances were 10 times to the positive instances (Panwar et al., 2013). For both balanced and realistic datasets, 20% of the data was picked randomly and kept as validation dataset, i.e., 36 positive and 36 negative examples for balanced and 36 positive and 356 negative examples for realistic dataset. The rest 80% of the data was used for training and 10-fold cross validation (Figure 1). The balanced dataset was used for the composition analysis, whereas, the prediction models were developed on both balanced as well as realistic dataset.

### Features extraction

#### Composition-based features

##### Amino acid composition

Amino acid composition (AAC) represents the fraction of each of the amino acids present in a given peptide/protein sequence. Each vector has 20 dimensions (20-D vector) representing the compositional frequency of 20 amino acids in the sequence. AAC has been widely used for binary/multiclass classification in several studies (Gupta et al., 2013a, 2014; Sharma et al., 2015). For AAC calculation only 20 naturally amino acids are considered. AAC can be calculated using the formula below.

$$\begin{array}{l} AAC\left(i\right)=\frac{\text{Total number of amino acid }\left(\text{i}\right)}{\text{Total number of all possible amino acids}}\times 100 \end{array}$$

where, *AAC(i)* is the amino acid composition of the amino acid (i) among all the 20 naturally occurring amino acids.

##### Dipeptide composition

Dipeptide composition (DPC) represents the total number of dipeptide divided by all the possible combinations of dipeptides present in the given protein/peptide sequence. These individual combinations of dipeptides collectively form an input vector of 400 dimensions (400-D vector) which includes all the possible dipeptides of 20 amino acids. DPC has also been widely used for binary/multiclass classification in several studies (Gupta et al., 2013b, 2014; Sharma et al., 2015). Compared to AAC, DPC provides additional information on the local arrangement of residues in a sequence. DPC can be calculated using the following formula.

$$\begin{array}{l} DPC\left(i\right)=\frac{\text{Total number of dipeptides }\left(\text{i}\right)}{\text{Total number of all possible dipeptides}}\times 100 \end{array}$$

where, *DPC(i)* is the dipeptide frequency of dipeptide (i) among all the possible 400 dipeptides.

#### Motif-based feature

Sequence motifs in a given protein/peptide sequence plays an important role in the functionality of the protein/peptide (Dhanda et al., 2013; Tompa et al., 2014). The conserved functional motifs have also been used for the functional annotation of amino acid sequences (ElHefnawi et al., 2011). Several studies have reported the presence of specific sequence motifs in BIPs which provide biofilm inhibitory properties to these peptide sequences (Dean et al., 2011). Therefore, the identification of exclusive motifs present in experimentally validated BIPs and their use in prediction methods is likely to help in the identification of novel BIPs. MERCI software was used for the identification of sequence motifs specific to BIPs (https://dtai.cs.kuleuven.be/software/merci; Vens et al., 2011). It is an online tool for the identification of exclusive motifs present in the positive dataset by comparing it with the negative dataset. The exclusive motif identification was carried out in a single step by utilizing the Betts-Russell algorithm, where, BIPs were considered as positive dataset and non-BIPs were considered as negative dataset.

#### Hybrid features

Several previous studies have shown that the combination of multiple features may provide better prediction accuracy (Saha and Raghava, 2006). Hence, the composition based features and motif based features were combined to create a comprehensive hybrid features set. In order to utilize the hybrid features, a weightage scheme was employed, where the weight of +0.5 was assigned to the AAC and DPC based SVM score if the exclusive positive motif was present in the given peptide sequence.

### Construction of machine learning based prediction models

#### Support vector machine

SVM was implemented by using SVM^light^ package available at http://svmlight.joachims.org/. This classification algorithm draws a hyperplane between positive and negative data, and uses this hyperplane for the classification. This hyperplane can be drawn via choosing multiple functions such as, linear, polynomial and radial basis. Multiple kernels and classification functions can be optimized to obtain the best classification performances. SVM has been used widely for the binary classification in various classification tools (Gupta et al., 2014).

#### Random Forest

Random Forest (RF) was implemented using randomForest package in R (http://cran.r-project.org//). RF is best suited for the analysis of large datasets because of its multi model classification algorithm (Sharma et al., 2015), high accuracy of prediction and the information of highly important variables for the classification (Touw et al., 2013; Chaudhary et al., 2015). At the time of training and optimization of RF, randomly selected ~66% of the data was used for training and rest of the data was considered as Out-of-bag (OOB) data for estimating the prediction accuracy. The optimization of number of randomly selected variables (mtry) for the classification at each node and number of classification models in the forest was carried out to obtain the lowest OOB error, i.e., highest accuracy.

#### Performance evaluation of machine learning models

Performance evaluation and comparison of machine learning methods is an important part of modeling. To evaluate the performance of any method, cross-validation technique is among the most widely used and accepted technique. In the cross validation technique, total data is divided into parts depending upon the folds (n-fold CV) selected. In case of 10-fold cross validation (CV-10) which has been used in this study, the data was divided into 10 parts, out of which 9 parts were used for the training, and the 10th part was used for the testing purpose. This process was repeated till all the parts were used at least once as test set, and the overall performance on the all 10 parts was evaluated and reported. Performance of the SVM and RF models has been measured by both threshold dependent as well as threshold independent parameters. AUC (threshold independent parameter) was calculated by using PERF software for SVM model and by using pROC package in R for RF model. Other parameters which are threshold dependent, such as sensitivity (Sen), specificity (Spec), accuracy (Acc), and Matthews's correlation coefficient (MCC) were calculated from the confusion matrix. The following equations were used for the calculation of these parameters:

$$\begin{array}{l} Accuracy=\frac{TP+TN}{TP+FN+FP+TN}\\ Sensitivity=\frac{TP}{TP+FN}\\ Specificity=\frac{TN}{TN+FP}\\ MCC=\frac{\left(TP\times TN\right)-\left(FP\times FN\right)}{\sqrt{\left(TP+FP\right)\left(TP+FN\right)\left(TN+FP\right)\left(TN+FN\right)}} \end{array}$$

where, *TP* = True Positive, *FP* = False Positive, *FN* = False Negative, *TN* = True Negative

#### Data on human gut and probiotic bacterial strains

All the protein sequences belonging to gut associated Lactobacillus and Bifidobacterium genus were obtained from Swiss-Prot. A total of 2471 protein sequences were obtained for different gut associated species of Lactobacillus and Bifidobacterium (*B. bifidum, B. adolescentis, B. infantis, B. longum, B. breve, L. fermentum, L. casei, L. paracasei, L. rhamnosus, L. johnsonii, L. plantarum*; Reuter, 2001; Grover et al., 2013). The retrieved protein sequences belonging to the above bacterial species were analyzed through the prediction pipeline, using the default prediction model and a window length of 12 amino acids at the web server. The top 20 peptides obtained in the study were also analyzed with the Similarity search module of web server to identify similar experimentally validated BIPs and the possible microorganism being inhibited.

## Results

Considering the prominent role of microbial biofilm in most of the chronic infections and their high resistance to the available therapeutics and host immune system, exploration of novel BIPs is highly desired. In this study, the sequence based features of the experimentally validated BIPs have been used to predict the biofilm inhibiting activity of query peptides. The experimentally validated BIPs from the database BaAMPs (www.baamps.it) were used as positive dataset and compared with randomly generated negative peptides from the Swiss-Prot protein sequence database. The detailed description methodology of the dataset creation and prediction model creation is described in Figure 1.

### Compositional analysis

**Figure 2:**
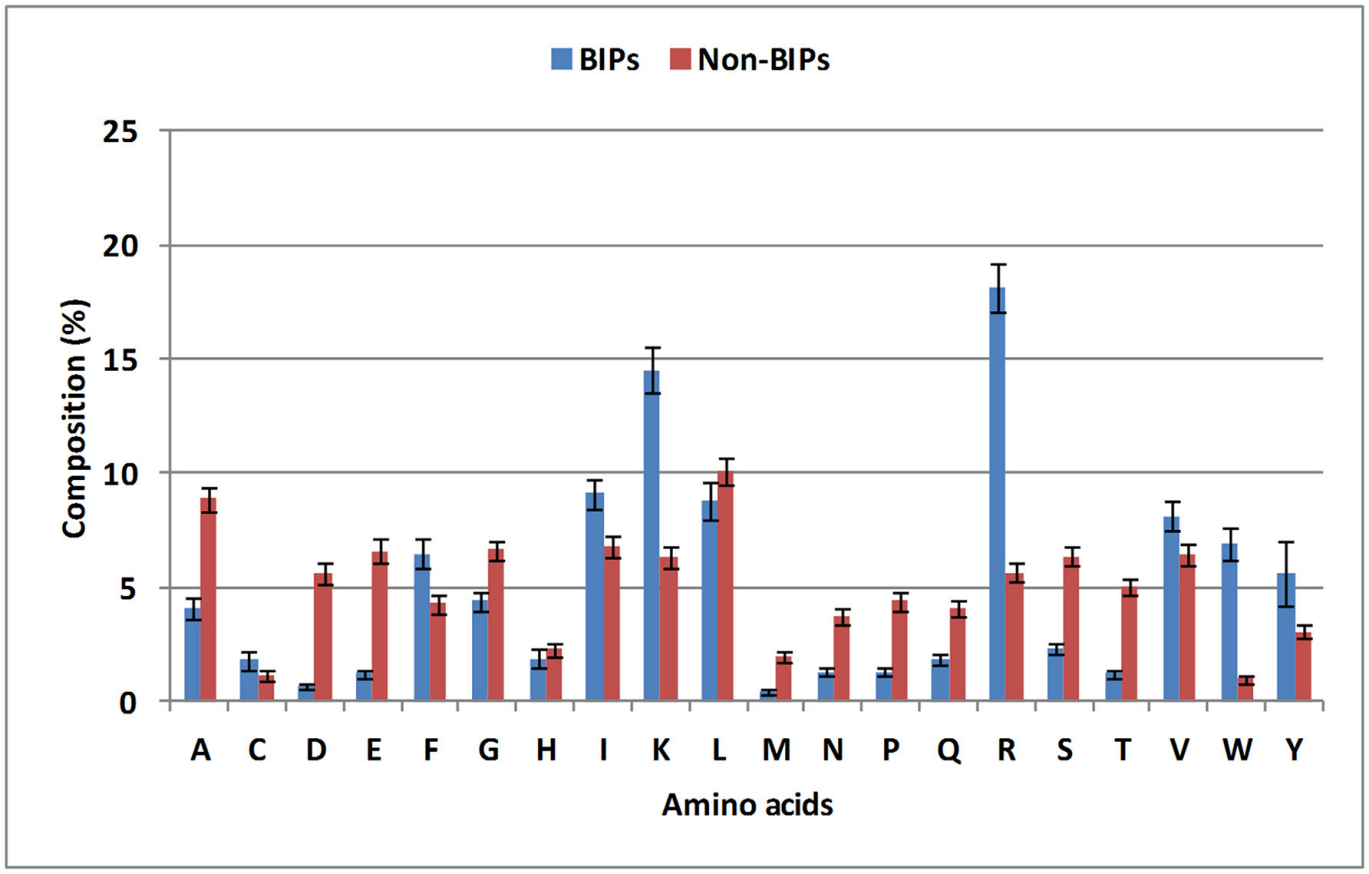
**Compositional analysis of biofilm inhibiting and biofilm non-inhibiting peptides**. Positively charged amino acids (Lys and Arg) and aromatic amino acids (Trp, Tyr, and Phe) were found to be abundant in BIPs, whereas, non-BIPs were rich in negatively charged amino acids, such as Glu and Asp.

To evaluate the distribution of amino acids in BIPs and non-BIPs, an amino acid compositional analysis was performed. BIPs were found to be abundant in positively charged amino acids (Lys and Arg) and aromatic amino acids (Trp, Tyr, and Phe), whereas, non-BIPs were rich in negatively charged amino acids (Glu and Asp) as shown in Figure 2, Supplementary Table S1. Aliphatic amino acids did not show any biasness and were almost equally abundant in BIPs and non-BIPs. Further, the dipeptide frequency/bias analysis was performed to detect the preferences of ordered dipeptides in the BIPs verses non-BIPs. Out of 400 different dipeptides, 183 were differentially present in BIPs and non-BIPs (Welch's *t*-test, *p* < 0.05). The 10 topmost differentiating dipeptides between the two sets were DE, EE, FK, IR, IV, KK, KR, LE, RI, RK (Supplementary Table S2), whereas, the top most abundant dipeptides in BIPs were YY, RI, IR, RW, RR, WR, KK, RV, VR, KR.

From this analysis, it is apparent that the most abundant dipeptides in BIPs are mostly the pairs of positively charged—aliphatic amino acids or positively charged—aromatic amino acid or aromatic—aromatic amino acids, whereas, the most abundant dipeptides in the non-BIPs are pairs of negatively charged—negatively charged amino acids or negatively charged—aliphatic amino acids or aliphatic—aliphatic amino acids. As expected, these results are well in agreement with the amino acids compositional analysis as the positively charged and aromatic amino acids were also found as most abundant in BIPs and the negatively charged amino acids were the most abundant in non-BIPs.

### Motif analysis

To identify the motifs present in BIPs, the positive training dataset was analyzed using MERCI software as mentioned in the Methods section. The overall coverage of motif represents the number of biofilm inhibiting peptides containing that particular motif. From the positive dataset, a total of 13 motifs were identified which were uniquely present in the biofilm inhibiting peptides. These motifs had the overall coverage ranging from 31 to 33 peptides out of the complete positive set of 178 peptide sequences (Supplementary Table S3). The “positive hydrophobic hydrophobic polar hydrophobic hydrophobic charged polar hydrophobic” and “positive hydrophobic hydrophobic polar hydrophobic hydrophobic positive polar hydrophobic” motifs were most abundant in the BIPs with the coverage value of 33 and 32, respectively (iSupplementary Table S3). All the motifs were rich in the positively charged amino acids and hydrophobic amino acids which corroborate well with the amino acid compositional analysis and dipeptide frequency analysis where the positively charged amino acids were most abundant in BIPs.

### Machine learning-based classification

The BIPs and non-BIPs were significantly different in amino acid sequence-based features (Figure 2). Hence, the compositional features of peptides sequences were used for the classification of peptide into BIPs or non-BIPs using machine learning approaches. Balanced and realistic datasets were used separately for the development of classification models using AAC and DPC features. Two kinds of machine learning [SVM and Random Forest (RF)] models were constructed based on the SVM and RF algorithms, and multiple parameters such as kernel value, mtry, ntree were optimized independently to achieve the best prediction performance measured as minimum OOB error. Since several models were developed using the two datasets (balanced and realistic), using AAC, DPC and hybrids as feature, and using SVM and Random Forest as machine learning technique; only outperforming SVM-based models are discussed in the manuscript. The performance of SVM-based model was better than the RF-based model (**Table 2**).

### Performance of SVM and RF using AAC and DPC as input features

There was a significant difference between the BIPs and non-BIPs with respect to amino acid composition, and hence, this difference was utilized for the classification (Figure 2). On the balanced dataset, SVM model with best performance was selected with rbf kernel (*t* = 2), gamma parameter (g) = 0.001, trade off factor (c) = 0.5 and a cost factor (j) of 4. This model displayed overall accuracy and MCC of 92.25% and 0.85, respectively. On the realistic dataset, the SVM model with best parameters was selected with the rbf kernel (*t* = 2), gamma parameter (g) = 0.001, trade off factor (c) = 10 and a cost factor (j) of 1. The selected SVM models showed the overall accuracy and MCC values of 97.64% and 0.86, respectively (Table 1). The threshold independent parameter, Area Under Curve (AUC), was also calculated to evaluate the performance of the different models. For the selected models, the values of 0.97 and 0.98 were respectively achieved on the balanced and realistic dataset (Table 1). These results suggest that amino acid composition is a good feature for the peptides binary classification as BIP or non-BIP with high accuracy.

**Table 1:** **Performance (CV-10 fold) of SVM-based models on both balanced and realistic datasets using AAC and DPC features as input**.

**Dataset**	**Feature**	**Kernel**	**Thr**	**Sen**	**Spec**	**Acc**	**MCC**	**AUC**	**Parameter**
Balanced dataset	AAC	t0	0.1	90.14	93.66	91.9	0.84	0.94	t:0,c:80
		t1	−0.1	86.62	96.48	91.55	0.84	0.96	t:1,d:2
		t2	0.2	93.66	90.85	92.25	0.85	0.97	g:0.001:c:0.5:j:4
	DPC	t0	−0.1	90.14	89.44	89.79	0.8	0.96	t:0,c:990
		t1	−0.2	90.85	94.37	92.61	0.85	0.96	t:1,d:1
		t2	0.1	84.51	95.77	90.14	0.81	0.96	g:0.001:c:1:j:1
Realistic dataset	AAC	t0	0.3	69.72	99.3	96.62	0.78	0.95	t:0,c:5
		t1	−0.6	88.03	98.38	97.45	0.85	0.98	t:1,d:3
		t2	−0.2	86.62	98.74	97.64	0.86	0.98	g:0.001:c:10:j:1
	DPC	t0	0.5	78.87	97.89	96.17	0.77	0.95	t:0,c:990
		t1	−0.3	79.58	98.67	96.93	0.81	0.96	t:0,d:1
		t2	−0.3	83.8	98.88	97.51	0.85	0.96	g:0.001:c:1:j:2

*The models constructed using balanced dataset displayed the highest MCC values of 0.85 for both AAC and DPC. The models constructed using realistic dataset displayed the highest MCC values of 0.86 and 0.85, respectively, on AAC and DPC as input features*.

The dipeptide composition features were utilized for preparing the SVM based classification models. On balanced dataset, the SVM model with best performance was selected with the polynomial kernel (*t* = 1) and parameter (*d* = 1). This model showed the overall accuracy and MCC values of 92.61% and 0.85, respectively. On realistic dataset, again the SVM model with best performance was selected with the rbf kernel (*t* = 2), gamma parameter (*g* = 0.001), trade off factor (*c* = 1) and a cost factor (j) of 2 which displayed the overall accuracy and MCC value of 97.51% and 0.85, respectively. AUC values for the best models on balanced and realistic dataset were 0.96 and 0.96, respectively (Table 1).

Performance of RF models was optimized at different mtry values at the time of classification using tuneRF function in R. For both balanced and realistic datasets, the best mtry was 8 for AAC features, and was 10 for DPC features, since at these mtry values the RF models showed the least OOB error or higher classification accuracy (Supplementary Figures S1A–D). The final RF models were constructed at the optimized mtry (value) and ntree (500) values, and similarly the final SVM models were constructed using optimized parameters. On realistic dataset, using AAC as the input feature, the SVM based model showed highest accuracy and MCC of 97.64% and 0.86, respectively, whereas, the RF model using AAC showed accuracy and MCC of 97.25 and 0.82, respectively. Similarly, using DPC as the input feature, RF model showed accuracy and MCC values of 96.04% and 0.73 respectively, which was lower than DPC-based SVM model. Overall, SVM prediction models performed better than RF models, and hence selected for further evaluation and validation (Tables 1, 2).

**Table 2:** **Performance (CV-10 fold) of RF models both balanced and realistic datasets using AAC and DPC features as input at optimized parameters**.

**Dataset**	**Features**	**Thr**	**Sen**	**Spec**	**Acc**	**MCC**	**AUC**	**Parameters**
Balanced dataset	AAC	0.5	89.44	94.37	91.90	0.84	0.96	mtry:8,ntree:500
	DPC	0.5	90.85	84.51	87.68	0.76	0.95	mtry:10,ntree:500
Realistic dataset	AAC	0.5	75.35	99.44	97.25	0.82	0.97	mtry:8,ntree:500
	DPC	0.5	58.45	99.79	96.04	0.73	0.95	mtry:10,ntree:500

*It is apparent that AAC-based RF models could achieve maximum MCC value of 0.84 with an accuracy of 91.9% and displayed better performance than DPC-based RF models*.

### Hybrid model

Although the SVM models based on the AAC and DPC features showed good performance, to further improve upon the performance, these features were used in the combination with unique motif feature to construct the hybrid SVM models (AAC_Motif and DPC_Motif hybrid models). On balanced dataset, the DPC_Motif (Accuracy = 95.4%, MCC = 0.91) performed better than AAC_Motif (Accuracy = 93.6%, MCC = 0.88). Furthermore, on the realistic dataset, the hybrid models based on AAC_Motif and DPC_Motif displayed the accuracy of 97.7 and 97.8, respectively, with an equal MCC = 0.87 for both models (Table 3).

**Table 3:** **Performance (CV-10 fold) of SVM-based models on both balanced and realistic datasets using the composition-motif hybrid features**.

**Dataset**	**Feature**	**Kernel**	**Thr**	**Sen**	**Spec**	**Acc**	**MCC**	**AUC**	**Parameter**
Balanced dataset	AAC_Motif hybrid	t0	0.3	91.55	94.37	92.96	0.86	0.95	t:0,c:60
		t1	0.2	90.14	97.18	93.66	0.88	0.95	t:1,d:1
		t2	0.9	90.14	97.18	93.66	0.88	0.97	g:0.001 c:0.05 j:4
	DPC_Motif hybrid	t0	0.4	87.32	95.07	91.2	0.83	0.97	t:0,c:990
		t1	−0.4	93.66	97.18	95.42	0.91	0.96	t:1,d:2
		t2	0.1	92.25	95.77	94.01	0.88	0.97	g:0.001 c:1 j:1
Realistic dataset	AAC_Motif hybrid	t0	0.3	75.35	99.3	97.13	0.82	0.96	t:0,c:5
		t1	−0.6	89.44	98.38	97.57	0.86	0.98	t:1,d:3
		t2	−0.3	88.73	98.67	97.77	0.87	0.98	g:0.001 c:4 j:1
	DPC_Motif hybrid	t0	0.5	78.87	97.89	96.17	0.77	0.95	t:0,c:990
		t1	−0.3	81.69	99.02	97.45	0.84	0.97	t:1,d:2
		t2	−0.3	85.92	99.02	97.83	0.87	0.97	g:0.001 c:2 j:1

*The models constructed using balanced dataset displayed the highest MCC values of 0.88 and 0.91 for AAC_Motif hybrid and DPC_Motif hybrid models, respectively. The models constructed using realistic dataset displayed the highest MCC values of 0.87 for both AAC_Motif hybrid and DPC_Motif Hybrid models*.

### Performance on validation dataset

Although, performance evaluation of machine learning techniques using 10-fold cross validation experiment is a well-accepted method, still there could be a possibility of over-fitting. Thus, further evaluation of final SVM models was carried out on a validation set of 20% peptides from each balanced and realistic dataset. On the balanced dataset, performance of the DPC_Motif model (MCC = 0.92) was higher as compared to the AAC_Motif model (MCC = 0.89), and on the realistic dataset, performance of the DPC_Motif model (MCC = 0.84) was higher as compared to the AAC_Motif model (MCC = 0.82) (Table 4). The performance of models on validation dataset shows that the models are free from over-fitting. Keeping realistic prediction in priority, the DPC_Motif hybrid model developed on realistic dataset was incorporated in the website for the prediction of BIPs.

**Table 4:** **Performance of composition-motif hybrid models on validation dataset**.

**Kernel**	**Thr**	**Sen**	**Spec**	**Acc**	**MCC**	**AUC**
**AAC-MOTIF HYBRID VALIDATION ON BALANCED DATASET**						
t0	0.3	94.44	86.11	90.28	0.81	0.96
t1	0.2	91.67	97.22	94.44	0.89	1
t2	0.9	91.67	97.22	94.44	0.89	0.99
**DPC-MOTIF HYBRID VALIDATION ON BALANCED DATASET**						
t0	0.4	88.89	94.44	91.67	0.83	0.99
t1	−0.4	91.67	100	95.83	0.92	1
t2	0.1	91.67	100	95.83	0.92	0.99
**AAC-MOTIF HYBRID VALIDATION ON REALISTIC DATASET**						
t0	0.3	72.22	98.03	95.66	0.73	0.98
t1	−0.6	97.22	96.07	96.17	0.81	0.99
t2	−0.3	91.67	97.19	96.68	0.82	0.99
**DPC-MOTIF HYBRID VALIDATION ON REALISTIC DATASET**						
t0	0.5	83.33	95.51	94.39	0.71	0.97
t1	−0.3	86.11	98.03	96.94	0.82	0.98
t2	−0.3	91.67	97.75	97.19	0.84	0.99

*DPC_Motif Hybrid models on validation set displayed the highest MCC values of 0.92 and 0.84 on balanced and realistic datasets, respectively*.

### Prediction of BIPs from human gut probiotic bacterial strains

To investigate the presence of BIPs in a real biological environment where biofilm formation are common, such as human gut, the proteins from gut associated species of genus *Lactobacillus* and *Bifidobacterium* (Material and Methods section) were analyzed through the web server. Several BIPs were found abundant in the proteins from these species, from which the top scoring 20 peptides are shown in Table 5. Among these 20 peptides, the peptides (AIKQVKKLFKKW, IKQVKKLFKKWG, and KQVKKLFKKWGW) were from Bacteriocin plantaricin-A protein belonging to a probiotic bacterium *Lactobacillus plantarum*. Furthermore, when these peptides were analyzed in Similarity Search module of webserver, several of these peptides displayed high similarity to BIPs effective against potentially pathogenic bacteria (Supplementary File 2), such as *Burkholderia pseudomallei, Pseudomonas aeruginosa, Streptococcus mutans CGMCC 1.2500 (Mouth bacterium), Staphylococcus aureus ATCC33591*, which suggest their potential ability to inhibit growth of such bacteria in the human gut.

**Table 5:** **Top scoring 20 peptides (12-mers) found in protein sequences belonging to different gut associated species of Lactobacillus and Bifidobacterium**.

**Peptide sequence**	**Uniprot**	**Protein**	**Species**	**Num. motifs**	**SVM score**
KKLFKVVKKRGI	Q74IG8	Peptide chain release factor 3	*Lactobacillus johnsonii* (strainCNCMI-12250)	4	0.63
IKQVKKLFKKWG	P80214	Bacteriocin plantaricin-A	*Lactobacillus plantarum* (strainATCCBAA-793)	1	0.51
AIKQVKKLFKKW	P80214	Bacteriocin plantaricin-A	*Lactobacillus plantarum* (strainATCCBAA-793)	1	0.49
TKKLFKVVKKRG	Q74IG8	Peptide chain release factor 3	*Lactobacillus johnsonii* (strainCNCMI-12250)	4	0.47
KKRIHELLRTLK	Q8G838	Putative ABC transporter ATP-binding protein BL0043	*Bifidobacterium longum* (strainNCC2705)	1	0.41
DRIKKAAKKIQN	Q74K31	Glucose-6-phosphate isomerase	*Lactobacillus johnsonii* (strainCNCMI-12250)	1	0.38
RIKKAAKKIQND	Q74K31	Glucose-6-phosphate isomerase	*Lactobacillus johnsonii* (strainCNCMI-12250)	1	0.38
KQVKKLFKKWGW	P80214	Bacteriocin plantaricin-A	*Lactobacillus plantarum* (strainATCCBAA-793)	1	0.37
QTKKLFKVVKKR	Q74IG8	Peptide chain release factor 3	*Lactobacillus johnsonii* (strainCNCMI-12250)	4	0.36
NRKKHVIRVCQD	Q8G3S4	tRNA(Ile)-lysidine synthase	*Bifidobacterium longum* (strainNCC2705)	2	0.35
NRKKHVIRVCQD	B7GP48	tRNA(Ile)-lysidine synthase	*Bifidobacterium longum* subsp. infantis (strainATCC15697)	2	0.35
NRKKHVIRVCQD	B3DR08	tRNA(Ile)-lysidine synthase	*Bifidobacterium longum* (strainDJO10A)	2	0.35
RIGDRVIRAARV	Q8G6W2	Protein Grp E	*Bifidobacterium longum* (strainNCC2705)	1	0.31
RIGDRVIRAARV	A1A3P4	Protein Grp E	*Bifidobacterium adolescentis* (strainATCC15703)	1	0.31
RKKHVIRVCQDG	Q8G3S4	tRNA(Ile)-lysidine synthase	*Bifidobacterium longum* (strainNCC2705)	5	0.30
RKKHVIRVCQDG	B7GP48	tRNA(Ile)-lysidine synthase	*Bifidobacterium longum* subsp. infantis (strainATCC15697)	5	0.30
RKKHVIRVCQDG	B3DR08	tRNA(Ile)-lysidine synthase	*Bifidobacterium longum* (strainDJO10A)	5	0.30
QAKKRIHELLRT	Q8G838	Putative ABC transporter ATP-bindingproteinBL0043	*Bifidobacterium longum* (strainNCC2705)	1	0.29
PAAVLLKKAAKV	P62435	50S ribosomal protein L11	*Lactobacillus johnsonii* (strainCNCMI-12250)	1	0.29
DKIVKKIFKKYS	P62471	Ribosomal RNA small subunit methyl transferase H	*Lactobacillus johnsonii* (strainCNCMI-12250)	2	0.26
IKKAYRKLSKKY	Q88VM1	Chaperone protein DnaJ	*Lactobacillus plantarum* (strainATCCBAA-793/NCIMB8826/WCFS1)	10	0.25
KIVKKIFKKYSE	P62471	Ribosomal RNA small subunit methyl transferase H	*Lactobacillus johnsonii* (strainCNCMI-12250)	4	0.24
AQAKKRIHELLR	Q8G838	Putative ABC transporter ATP-binding protein BL0043	*Bifidobacterium longum* (strainNCC2705)	1	0.23
AKKRIHELLRTL	Q8G838	Putative ABC transporter ATP-binding protein BL0043	*Bifidobacterium longum* (strainNCC2705)	1	0.22
EDKIVKKIFKKY	P62471	Ribosomal RNA small subunit methyl transferase H	*Lactobacillus johnsonii* (strainCNCMI-12250)	2	0.19

### Web server

A web server tool has been developed to facilitate the identification of biofilm inhibitory potential of a query peptide/protein which could be useful in the discovery of novel BIPs that can act as lead peptides for experimental validation. The prediction model is freely available as a web server at http://metagenomics.iiserb.ac.in/biofin/ and http://metabiosys.iiserb.ac.in/biofin/. The idifferent modules of the web server are explained below.

### Peptide prediction

This module of web server is designed for the submission of single or multiple peptide sequences (4–45 amino acids long) in FASTA format. Query sequence will pass through the prediction pipeline where the DPC-motif hybrid SVM model will predict if the query peptide has any biofilm inhibitory activity. Further, to make more stringent prediction, the prediction threshold option can be used. Virtual screening and designing option has also been provided, which allows user to look at the result table, modify the query peptides and resubmit the selected peptides on the basis of their prediction score. This option allows for the substitution of each amino acid of the peptide with other amino acids. Resubmission after the substitution will again provide the results in the same tabular format with prediction scores. It will allow the users to predict the biofilm inhibitory nature in the multiple variants of the query peptide, and hence, will be useful in understanding the position specific effects of each amino acid in modulating the biofilm inhibitory activity of the peptide.

### Protein scan

This module can be used to identify the sequence regions (peptides) in a protein sequence which may potentially inhibit biofilm formation. The user can also select the desired length of the peptide to be considered for prediction. This module will results in generation of multiple peptides of desired length which will pass through the same prediction pipeline along with virtual screening and provide results in the tabular format.

### Peptide mapping

This module has been developed to allow the users to map all the experimentally validated biofilm inhibitory peptides on the query sequence. Using the module, user can align the 100% identical BIPs on the query peptide/protein sequences which are also provided with the other bioassay related information.

### Similarity search

This module provides an option to perform the Smith-Waterman homology search of query sequence against the experimentally validated BIPs. The top hits obtained are shown with complete local alignment and corresponding scores.

## Discussion

In this work, we have described a computational method to predict the biofilm inhibiting peptides. Since the molecular function and the corresponding biological activity of peptides and proteins can be predicted computationally using sequence data (Lee et al., 2007), we have used the available sequence data of BIPs to develop this tool for the prediction of biofilm inhibiting peptides. From amino acid and dipeptide frequency compositional analysis, it is apparent that biofilm inhibiting peptides have preference for the positively charged and aromatic amino acids. Reports have also shown that the peptides rich in Lys and Leu show potential biofilm inhibitory activity (Segev-Zarko et al., 2015). The positively charged amino acids form a “charge clamp” which help in the proper association of these biofilm inhibitory peptides with their targets. The positive charge further help in association of these peptides with the microbial cell membrane (Bahar and Ren, 2013). For example, replacing the lysines of VQDLL with acidic amino acids reduces the biofilm inhibitory activity of the VQDLL against Mfa1 target of *Porphyromonas gingivalis* (Daep et al., 2008). These peptides show biofilm inhibiting activity mainly by inhibiting the quorum sensing and interfere with the microbial cell adhesion to the surface and other microbial cells by coating either microbial cells or surface or both.

The microbial cell-cell adhesion and cell-surface adhesion is highly dependent on the cell adhesion surface proteins and hence, these proteins can be a good target for biofilm inhibition. These proteins usually recognize specific sequence motifs forming a specific secondary structure, e.g., minor fimbrial antigen (Mfa1) of *Porphyromonas gingivalis* important in formation of oral biofilm, bind to specific motifs such as NITVK and KKVQDLLKK forming an alpha helical structure. From our motif analysis using MERCI, we have identified the most abundant motif in biofilm inhibiting peptides to be “positive hydrophobic hydrophobic polar hydrophobic hydrophobic charged polar hydrophobic.”

Preparing a prediction method for biofilm inhibiting peptides was challenging because only a limited number of experimentally validated BIPs are known, and also because these peptides show a lot of variation in sequence and length. However, using the amino acid composition and dipeptide frequency as features to make the fix length vectors for SVM training appears a successful approach for the prediction of BIPs. Since the BIPs are significantly abundant in certain amino acids and dipeptides, almost every model displayed greater than 90% accuracy. Although, RF-based models displayed good performance, the SVM-based models which outperformed the RF-based models were considered for further evaluation and integration of biological motifs. Furthermore, the incorporation of motif information as weightage in DPC-based SVM models made it biologically more relevant and efficient, since it considerably enhanced the performance of the models. As apparent, the performance of models on validation dataset attests that the good performance is not due to over optimization.

This study on balanced as well as on realistic datasets ensures that in realistic situation, where, the chances of negative examples are higher, the models would perform well. The case study on different gut associated species of *Lactobacillus* and *Bifidobacterium* supports the potential of this method and web server for identification of BIPs. We anticipate that the tools provided in the web server will be very helpful in the discovery and designing of novel biofilm inhibiting peptides.

## Authors contribution

SG developed SVM-based models. AS developed Random Forest models. SG and SJ developed web server. VS and SG conceived the work, participated in the design of the study. AS, SJ, SG, and VS drafted the manuscript. All authors read and approved the final manuscript.

### Conflict of interest statement

The authors declare that the research was conducted in the absence of any commercial or financial relationships that could be construed as a potential conflict of interest.

## Supplementary material

Supplementary Figure S1 **Optimization of Random Forest parameters using AAC and DPC**. For both balanced and realistic datasets, the best mtry value was 8 for AAC as the input feature and was 10 for DPC as the input feature, where the least OOB error was achieved.

Supplementary Table S1 **Amino acid composition distribution between biofilm-inhibiting and biofilm non-inhibiting peptides**.

Supplementary Table S2 **Dipeptide composition distribution between biofilm-inhibiting and biofilm non-inhibiting peptides**.

Supplementary Table S3 **MERCI motifs extracted from biofilm-inhibiting and biofilm non-inhibiting peptides**.

Supplementary Table S4 **Microbial species assayed as positive against biofilm inhibiting peptides in BaAMP database**.

## Abbreviations

AAC: Amino acid composition
Acc: Accuracy
AUC: Area under curve
BIPs: Biofilm inhibiting peptides
DPC: Dipeptide composition
MCC: Matthews correlation coefficient
non-BIPs: Biofilm non-inhibiting peptides
OOB: Out-of-bag
PHY: Physiochemical properties
RF: Random Forest
Sen: Sensitivity
Spec: Specificity
SVM: Support Vector Machine.
