# Supplementary figures and images for: Prediction of Biofilm Inhibiting Peptides: An In silico Approach
Source: Front Microbiol. 2016 Jun 16;7:949. doi: 10.3389/fmicb.2016.00949 (PMC4909740; doi:10.3389/fmicb.2016.00949)

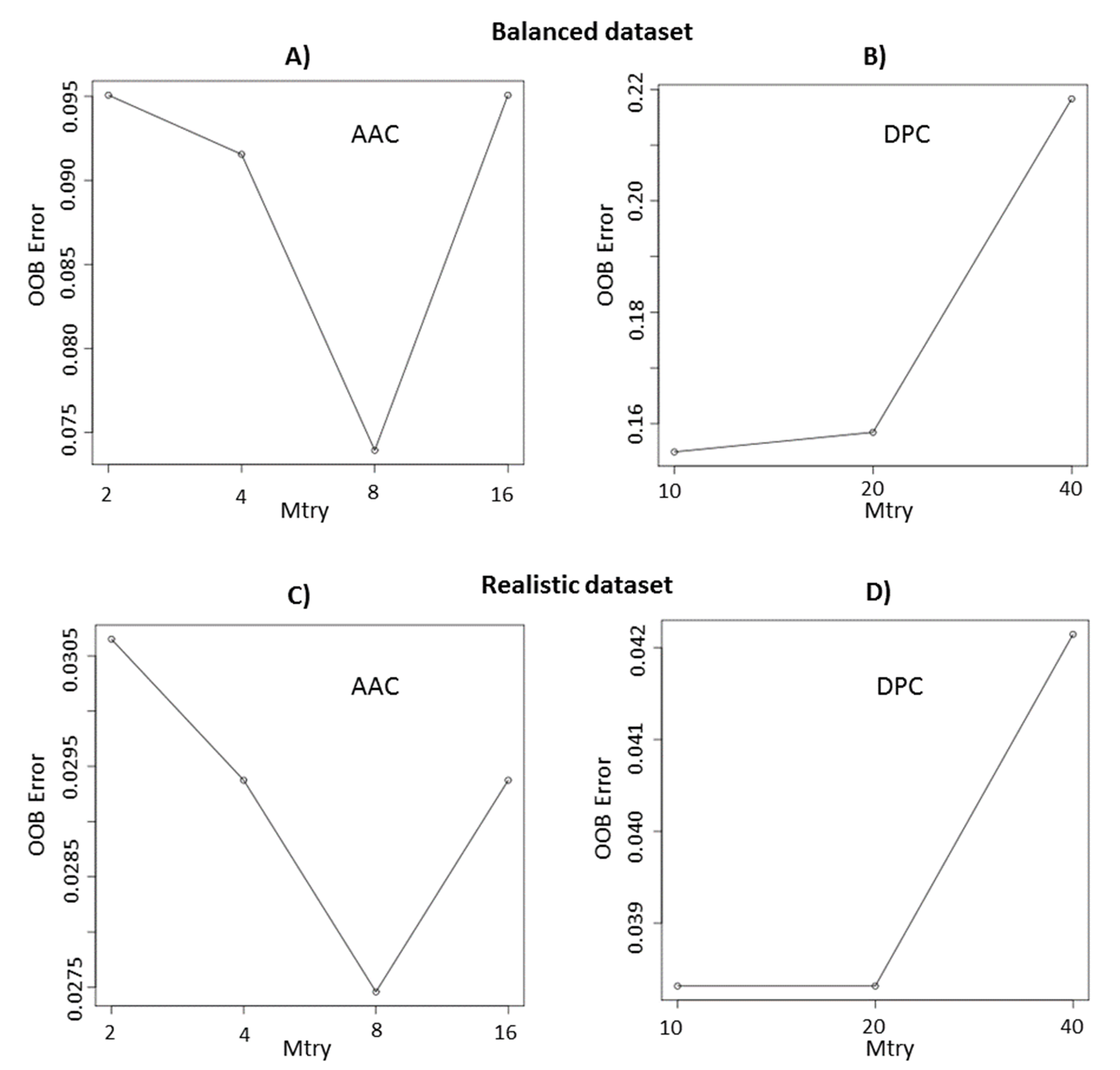

Supplement: Supplementary Figure S1 — Optimization of Random Forest parameters using AAC and DPC. For both balanced and realistic datasets, the best mtry value was 8 for AAC as the input feature and was 10 for DPC as the input feature, where the least OOB error was achieved. [file Image1.TIF]
