# Supplementary material for: Prediction of Biofilm Inhibiting Peptides: An In silico Approach
Source: Front Microbiol. 2016 Jun 16;7:949. doi: 10.3389/fmicb.2016.00949 (PMC4909740; doi:10.3389/fmicb.2016.00949)
Supplement: Supplementary file 3 [file DataSheet2.DOC]

**Supplementary file 2**

**Results of similarity search of top scoring 20 peptides found in gut probiotic bacteria.**

**List of peptides submitted:**

>Pep1

AIKQVKKLFKKW

>Pep2

AKKRIHELLRTL

>Pep3

AQAKKRIHELLR

>Pep4

DKIVKKIFKKYS

>Pep5

DRIKKAAKKIQN

>Pep6

EDKIVKKIFKKY

>Pep7

IKKAYRKLSKKY

>Pep8

IKQVKKLFKKWG

>Pep9

KIVKKIFKKYSE

>Pep10

KKLFKVVKKRGI

>Pep11

KKRIHELLRTLK

>Pep12

KQVKKLFKKWGW

>Pep13

NRKKHVIRVCQD

>Pep14

PAAVLLKKAAKV

>Pep15

QAKKRIHELLRT

>Pep16

QTKKLFKVVKKR

>Pep17

RIGDRVIRAARV

>Pep18

RIKKAAKKIQND

>Pep19

RKKHVIRVCQDG

>Pep20

TKKLFKVVKKRG

**Similarity search results obtained from BIOFIN web server:**

1>>>Pep1 - 12 aa

Library: BIOFIN/ssearch-baamp/biofin 15265 residues in 786 sequences

opt E()

< 20 0 0:

22 0 0: one = represents 2 library sequences

24 0 0:

26 0 0:

28 0 0:

30 1 1:*

32 32 4:=*==============

34 17 11:=====*===

36 35 23:===========*======

38 6 39:=== *

40 26 54:============= *

42 74 66:================================*====

44 52 72:========================== *

46 41 74:===================== *

48 94 71:===================================*===========

50 69 65:================================*==

52 104 57:============================*=======================

54 56 48:=======================*====

56 39 40:===================*

58 9 33:===== *

60 46 27:=============*=========

62 13 22:======= *

64 4 17:== *

66 8 14:==== *

68 9 11:=====*

70 6 8:===*

72 3 7:== *

74 5 5:==*

76 18 4:=*=======

78 0 3: *

80 0 2:*

82 15 2:*=======

84 4 1:*=

86 0 1:*

88 0 1:*

90 0 1:*

92 0 1:*

94 0 0:

96 0 0:

98 0 0:

100 0 0:

102 0 0:

104 0 0:

106 0 0:

108 0 0:

110 0 0:

112 0 0:

114 0 0:

116 0 0:

118 0 0:

>120 0 0:

15265 residues in 786 sequences

Statistics: MLE_cen statistics: Lambda= 0.1940; K=0.1304 (cen=39)

Kolmogorov-Smirnov statistic: 0.0769 (N=25) at 46

Algorithm: Smith-Waterman (SSE2, Michael Farrar 2006) (6.0 Mar 2007)

Parameters: BL50 matrix (15:-5), open/ext: -10/-2

Scan time: 0.000

The best scores are: s-w bits E(786)

[BaAMP:537](http://www.baamps.it/browse?task=peptide.display&ID=537)  pepti ( 11) 40 14.1 5.8

[BaAMP:540](http://www.baamps.it/browse?task=peptide.display&ID=540)  pepti ( 11) 40 14.1 5.8

[BaAMP:536](http://www.baamps.it/browse?task=peptide.display&ID=536)  pepti ( 11) 40 14.1 5.8

[BaAMP:538](http://www.baamps.it/browse?task=peptide.display&ID=538)  pepti ( 11) 40 14.1 5.8

[BaAMP:542](http://www.baamps.it/browse?task=peptide.display&ID=542)  pepti ( 11) 40 14.1 5.8

[BaAMP:537](http://www.baamps.it/browse?task=peptide.display&ID=537)  pepti ( 11) 40 14.1 5.8

[BaAMP:159](http://www.baamps.it/browse?task=peptide.display&ID=159)  pepti ( 26) 44 15.3 6.3

[BaAMP:13](http://www.baamps.it/browse?task=peptide.display&ID=13)  peptide ( 26) 44 15.3 6.3

[BaAMP:13](http://www.baamps.it/browse?task=peptide.display&ID=13)  peptide ( 26) 44 15.3 6.3

[BaAMP:160](http://www.baamps.it/browse?task=peptide.display&ID=160)  pepti ( 26) 44 15.3 6.3

[BaAMP:160](http://www.baamps.it/browse?task=peptide.display&ID=160)  pepti ( 26) 44 15.3 6.3

[BaAMP:4](http://www.baamps.it/browse?task=peptide.display&ID=4)  peptide, ( 26) 44 15.3 6.3

[BaAMP:7](http://www.baamps.it/browse?task=peptide.display&ID=7)  peptide, ( 26) 44 15.3 6.3

[BaAMP:159](http://www.baamps.it/browse?task=peptide.display&ID=159)  pepti ( 26) 44 15.3 6.3

[BaAMP:15](http://www.baamps.it/browse?task=peptide.display&ID=15)  peptide ( 26) 44 15.3 6.3

[BaAMP:15](http://www.baamps.it/browse?task=peptide.display&ID=15)  peptide ( 26) 44 15.3 6.3

[BaAMP:14](http://www.baamps.it/browse?task=peptide.display&ID=14)  peptide ( 26) 44 15.3 6.3

[BaAMP:14](http://www.baamps.it/browse?task=peptide.display&ID=14)  peptide ( 26) 44 15.3 6.3

[BaAMP:150](http://www.baamps.it/browse?task=peptide.display&ID=150)  pepti ( 26) 44 15.3 6.3

>>[BaAMP:537](http://www.baamps.it/browse?task=peptide.display&ID=537)  peptide, (11 aa)

s-w opt: 40 Z-score: 83.8 bits: 14.1 E(): 5.8

Smith-Waterman score: 40; 66.7% identity (100.0% similar) in 9 aa overlap (3-11:1-9)

10

Pep1 AIKQVKKLFKKW

:..::.:::

#BIOFI KRAKKFFKKLK

10

>>[BaAMP:540](http://www.baamps.it/browse?task=peptide.display&ID=540)  peptide, (11 aa)

s-w opt: 40 Z-score: 83.8 bits: 14.1 E(): 5.8

Smith-Waterman score: 40; 66.7% identity (100.0% similar) in 9 aa overlap (3-11:1-9)

10

Pep1 AIKQVKKLFKKW

:..::.:::

#BIOFI KRAKKFFKKLK

10

>>[BaAMP:536](http://www.baamps.it/browse?task=peptide.display&ID=536)  peptide, (11 aa)

s-w opt: 40 Z-score: 83.8 bits: 14.1 E(): 5.8

Smith-Waterman score: 40; 66.7% identity (100.0% similar) in 9 aa overlap (3-11:1-9)

10

Pep1 AIKQVKKLFKKW

:..::.:::

#BIOFI KRAKKFFKKLK

10

>>[BaAMP:538](http://www.baamps.it/browse?task=peptide.display&ID=538)  peptide, (11 aa)

s-w opt: 40 Z-score: 83.8 bits: 14.1 E(): 5.8

Smith-Waterman score: 40; 66.7% identity (100.0% similar) in 9 aa overlap (3-11:1-9)

10

Pep1 AIKQVKKLFKKW

:..::.:::

#BIOFI KRAKKFFKKPK

10

>>[BaAMP:542](http://www.baamps.it/browse?task=peptide.display&ID=542)  peptide, (11 aa)

s-w opt: 40 Z-score: 83.8 bits: 14.1 E(): 5.8

Smith-Waterman score: 40; 66.7% identity (100.0% similar) in 9 aa overlap (3-11:1-9)

10

Pep1 AIKQVKKLFKKW

:..::.:::

#BIOFI KRAKKFFKKPK

10

>>[BaAMP:537](http://www.baamps.it/browse?task=peptide.display&ID=537)  peptide, (11 aa)

s-w opt: 40 Z-score: 83.8 bits: 14.1 E(): 5.8

Smith-Waterman score: 40; 66.7% identity (100.0% similar) in 9 aa overlap (3-11:1-9)

10

Pep1 AIKQVKKLFKKW

:..::.:::

#BIOFI KRAKKFFKKLK

10

>>[BaAMP:159](http://www.baamps.it/browse?task=peptide.display&ID=159)  peptide, (26 aa)

s-w opt: 44 Z-score: 83.2 bits: 15.3 E(): 6.3

Smith-Waterman score: 44; 77.8% identity (88.9% similar) in 9 aa overlap (3-11:8-16)

10

Pep1 AIKQVKKLFKKW

:. ::::::

#BIOFI GRFKRFRKKFKKLFKKLSPVIPLLHL

10 20

>>[BaAMP:13](http://www.baamps.it/browse?task=peptide.display&ID=13)  peptide, Co (26 aa)

s-w opt: 44 Z-score: 83.2 bits: 15.3 E(): 6.3

Smith-Waterman score: 44; 77.8% identity (88.9% similar) in 9 aa overlap (3-11:8-16)

10

Pep1 AIKQVKKLFKKW

:. ::::::

#BIOFI GRFKRFRKKFKKLFKKLSPVIPLLHL

10 20

>>[BaAMP:13](http://www.baamps.it/browse?task=peptide.display&ID=13)  peptide, Co (26 aa)

s-w opt: 44 Z-score: 83.2 bits: 15.3 E(): 6.3

Smith-Waterman score: 44; 77.8% identity (88.9% similar) in 9 aa overlap (3-11:8-16)

10

Pep1 AIKQVKKLFKKW

:. ::::::

#BIOFI GRFKRFRKKFKKLFKKLSPVIPLLHL

10 20

>>[BaAMP:160](http://www.baamps.it/browse?task=peptide.display&ID=160)  peptide, (26 aa)

s-w opt: 44 Z-score: 83.2 bits: 15.3 E(): 6.3

Smith-Waterman score: 44; 77.8% identity (88.9% similar) in 9 aa overlap (3-11:8-16)

10

Pep1 AIKQVKKLFKKW

:. ::::::

#BIOFI GRFKRFRKKFKKLFKKLSPVIPLLHL

10 20

>>[BaAMP:160](http://www.baamps.it/browse?task=peptide.display&ID=160)  peptide, (26 aa)

s-w opt: 44 Z-score: 83.2 bits: 15.3 E(): 6.3

Smith-Waterman score: 44; 77.8% identity (88.9% similar) in 9 aa overlap (3-11:8-16)

10

Pep1 AIKQVKKLFKKW

:. ::::::

#BIOFI GRFKRFRKKFKKLFKKLSPVIPLLHL

10 20

>>[BaAMP:4](http://www.baamps.it/browse?task=peptide.display&ID=4)  peptide, Conc (26 aa)

s-w opt: 44 Z-score: 83.2 bits: 15.3 E(): 6.3

Smith-Waterman score: 44; 77.8% identity (88.9% similar) in 9 aa overlap (3-11:8-16)

10

Pep1 AIKQVKKLFKKW

:. ::::::

#BIOFI GRFKRFRKKFKKLFKKLSPVIPLLHL

10 20

>>[BaAMP:7](http://www.baamps.it/browse?task=peptide.display&ID=7)  peptide, Conc (26 aa)

s-w opt: 44 Z-score: 83.2 bits: 15.3 E(): 6.3

Smith-Waterman score: 44; 77.8% identity (88.9% similar) in 9 aa overlap (3-11:8-16)

10

Pep1 AIKQVKKLFKKW

:. ::::::

#BIOFI GRFKRFRKKFKKLFKKLSPVIPLLHL

10 20

>>[BaAMP:159](http://www.baamps.it/browse?task=peptide.display&ID=159)  peptide, (26 aa)

s-w opt: 44 Z-score: 83.2 bits: 15.3 E(): 6.3

Smith-Waterman score: 44; 77.8% identity (88.9% similar) in 9 aa overlap (3-11:8-16)

10

Pep1 AIKQVKKLFKKW

:. ::::::

#BIOFI GRFKRFRKKFKKLFKKLSPVIPLLHL

10 20

>>[BaAMP:15](http://www.baamps.it/browse?task=peptide.display&ID=15)  peptide, Co (26 aa)

s-w opt: 44 Z-score: 83.2 bits: 15.3 E(): 6.3

Smith-Waterman score: 44; 77.8% identity (88.9% similar) in 9 aa overlap (3-11:8-16)

10

Pep1 AIKQVKKLFKKW

:. ::::::

#BIOFI GRFKRFRKKFKKLFKKLSPVIPLLHL

10 20

>>[BaAMP:15](http://www.baamps.it/browse?task=peptide.display&ID=15)  peptide, Co (26 aa)

s-w opt: 44 Z-score: 83.2 bits: 15.3 E(): 6.3

Smith-Waterman score: 44; 77.8% identity (88.9% similar) in 9 aa overlap (3-11:8-16)

10

Pep1 AIKQVKKLFKKW

:. ::::::

#BIOFI GRFKRFRKKFKKLFKKLSPVIPLLHL

10 20

>>[BaAMP:14](http://www.baamps.it/browse?task=peptide.display&ID=14)  peptide, Co (26 aa)

s-w opt: 44 Z-score: 83.2 bits: 15.3 E(): 6.3

Smith-Waterman score: 44; 77.8% identity (88.9% similar) in 9 aa overlap (3-11:8-16)

10

Pep1 AIKQVKKLFKKW

:. ::::::

#BIOFI GRFKRFRKKFKKLFKKLSPVIPLLHL

10 20

>>[BaAMP:14](http://www.baamps.it/browse?task=peptide.display&ID=14)  peptide, Co (26 aa)

s-w opt: 44 Z-score: 83.2 bits: 15.3 E(): 6.3

Smith-Waterman score: 44; 77.8% identity (88.9% similar) in 9 aa overlap (3-11:8-16)

10

Pep1 AIKQVKKLFKKW

:. ::::::

#BIOFI GRFKRFRKKFKKLFKKLSPVIPLLHL

10 20

>>[BaAMP:150](http://www.baamps.it/browse?task=peptide.display&ID=150)  peptide, (26 aa)

s-w opt: 44 Z-score: 83.2 bits: 15.3 E(): 6.3

Smith-Waterman score: 44; 77.8% identity (88.9% similar) in 9 aa overlap (3-11:8-16)

10

Pep1 AIKQVKKLFKKW

:. ::::::

#BIOFI GRFKRFRKKFKKLFKKLSPVIPLLHL

10 20

2>>>Pep2 - 12 aa

Library: BIOFIN/ssearch-baamp/biofin 15265 residues in 786 sequences

opt E()

< 20 0 0:

22 0 0: one = represents 3 library sequences

24 0 0:

26 0 0:

28 0 0:

30 1 1:*

32 4 4:=*

34 14 11:===*=

36 25 23:=======*=

38 28 39:========== *

40 79 54:=================*=========

42 93 66:=====================*=========

44 52 72:================== *

46 93 74:========================*======

48 121 71:=======================*=================

50 34 65:============ *

52 18 57:====== *

54 27 48:========= *

56 14 40:===== *

58 16 33:====== *

60 33 27:========*==

62 13 22:===== *

64 13 17:=====*

66 2 14:= *

68 7 11:===*

70 71 8:==*=====================

72 21 7:==*====

74 3 5:=*

76 4 4:=*

78 0 3:*

80 0 2:*

82 0 2:*

84 0 1:*

86 0 1:*

88 0 1:* inset = represents 1 library sequences

90 0 1:*

92 0 1:* :*

94 0 0: *

96 0 0: *

98 0 0: *

100 0 0: *

102 0 0: *

104 0 0: *

106 0 0: *

108 0 0: *

110 0 0: *

112 0 0: *

114 0 0: *

116 0 0: *

118 0 0: *

>120 0 0: *

15265 residues in 786 sequences

Statistics: MLE_cen statistics: Lambda= 0.2138; K=0.1221 (cen=39)

Kolmogorov-Smirnov statistic: 0.1207 (N=23) at 48

Algorithm: Smith-Waterman (SSE2, Michael Farrar 2006) (6.0 Mar 2007)

Parameters: BL50 matrix (15:-5), open/ext: -10/-2

Scan time: 0.000

!! No sequences with E() < 10.000000

3>>>Pep3 - 12 aa

Library: BIOFIN/ssearch-baamp/biofin 15265 residues in 786 sequences

opt E()

< 20 0 0:

22 0 0: one = represents 3 library sequences

24 0 0:

26 0 0:

28 5 0:==

30 0 1:*

32 10 4:=*==

34 7 11:===*

36 29 23:=======*==

38 20 39:======= *

40 66 54:=================*====

42 128 66:=====================*=====================

44 44 72:=============== *

46 100 74:========================*=========

48 72 71:=======================*

50 46 65:================ *

52 25 57:========= *

54 9 48:=== *

56 44 40:=============*=

58 25 33:========= *

60 10 27:==== *

62 22 22:=======*

64 8 17:=== *

66 32 14:====*======

68 73 11:===*=====================

70 4 8:==*

72 5 7:==*

74 0 5: *

76 2 4:=*

78 0 3:*

80 0 2:*

82 0 2:*

84 0 1:*

86 0 1:*

88 0 1:* inset = represents 1 library sequences

90 0 1:*

92 0 1:* :*

94 0 0: *

96 0 0: *

98 0 0: *

100 0 0: *

102 0 0: *

104 0 0: *

106 0 0: *

108 0 0: *

110 0 0: *

112 0 0: *

114 0 0: *

116 0 0: *

118 0 0: *

>120 0 0: *

15265 residues in 786 sequences

Statistics: MLE_cen statistics: Lambda= 0.2494; K= 0.179 (cen=39)

Kolmogorov-Smirnov statistic: 0.0850 (N=22) at 42

Algorithm: Smith-Waterman (SSE2, Michael Farrar 2006) (6.0 Mar 2007)

Parameters: BL50 matrix (15:-5), open/ext: -10/-2

Scan time: 0.000

!! No sequences with E() < 10.000000

4>>>Pep4 - 12 aa

Library: BIOFIN/ssearch-baamp/biofin 15265 residues in 786 sequences

opt E()

< 20 0 0:

22 0 0: one = represents 3 library sequences

24 0 0:

26 0 0:

28 0 0:

30 0 1:*

32 19 4:=*=====

34 19 11:===*===

36 41 23:=======*======

38 18 39:====== *

40 48 54:================ *

42 59 66:==================== *

44 37 72:============= *

46 89 74:========================*=====

48 55 71:=================== *

50 133 65:=====================*=======================

52 62 57:==================*==

54 47 48:===============*

56 31 40:=========== *

58 10 33:==== *

60 18 27:====== *

62 12 22:==== *

64 25 17:=====*===

66 12 14:====*

68 4 11:== *

70 4 8:==*

72 2 7:= *

74 1 5:=*

76 3 4:=*

78 22 3:*=======

80 15 2:*====

82 0 2:*

84 0 1:*

86 0 1:*

88 0 1:* inset = represents 1 library sequences

90 0 1:*

92 0 1:* :*

94 0 0: *

96 0 0: *

98 0 0: *

100 0 0: *

102 0 0: *

104 0 0: *

106 0 0: *

108 0 0: *

110 0 0: *

112 0 0: *

114 0 0: *

116 0 0: *

118 0 0: *

>120 0 0: *

15265 residues in 786 sequences

Statistics: MLE_cen statistics: Lambda= 0.2011; K=0.1074 (cen=39)

Kolmogorov-Smirnov statistic: 0.0555 (N=25) at 52

Algorithm: Smith-Waterman (SSE2, Michael Farrar 2006) (6.0 Mar 2007)

Parameters: BL50 matrix (15:-5), open/ext: -10/-2

Scan time: 0.000

The best scores are: s-w bits E(786)

[BaAMP:13](http://www.baamps.it/browse?task=peptide.display&ID=13)  peptide ( 26) 40 14.8 8.4

[BaAMP:13](http://www.baamps.it/browse?task=peptide.display&ID=13)  peptide ( 26) 40 14.8 8.4

[BaAMP:14](http://www.baamps.it/browse?task=peptide.display&ID=14)  peptide ( 26) 40 14.8 8.4

[BaAMP:14](http://www.baamps.it/browse?task=peptide.display&ID=14)  peptide ( 26) 40 14.8 8.4

[BaAMP:150](http://www.baamps.it/browse?task=peptide.display&ID=150)  pepti ( 26) 40 14.8 8.4

[BaAMP:159](http://www.baamps.it/browse?task=peptide.display&ID=159)  pepti ( 26) 40 14.8 8.4

[BaAMP:159](http://www.baamps.it/browse?task=peptide.display&ID=159)  pepti ( 26) 40 14.8 8.4

[BaAMP:15](http://www.baamps.it/browse?task=peptide.display&ID=15)  peptide ( 26) 40 14.8 8.4

[BaAMP:15](http://www.baamps.it/browse?task=peptide.display&ID=15)  peptide ( 26) 40 14.8 8.4

[BaAMP:160](http://www.baamps.it/browse?task=peptide.display&ID=160)  pepti ( 26) 40 14.8 8.4

[BaAMP:160](http://www.baamps.it/browse?task=peptide.display&ID=160)  pepti ( 26) 40 14.8 8.4

[BaAMP:4](http://www.baamps.it/browse?task=peptide.display&ID=4)  peptide, ( 26) 40 14.8 8.4

[BaAMP:7](http://www.baamps.it/browse?task=peptide.display&ID=7)  peptide, ( 26) 40 14.8 8.4

[BaAMP:215](http://www.baamps.it/browse?task=peptide.display&ID=215)  pepti ( 20) 38 14.2 9.7

[BaAMP:216](http://www.baamps.it/browse?task=peptide.display&ID=216)  pepti ( 20) 38 14.2 9.7

>>[BaAMP:13](http://www.baamps.it/browse?task=peptide.display&ID=13)  peptide, Co (26 aa)

s-w opt: 40 Z-score: 80.8 bits: 14.8 E(): 8.4

Smith-Waterman score: 40; 63.6% identity (72.7% similar) in 11 aa overlap (2-12:8-18)

10

Pep4 DKIVKKIFKKYS

: ::.::: :

#BIOFI GRFKRFRKKFKKLFKKLSPVIPLLHL

10 20

>>[BaAMP:13](http://www.baamps.it/browse?task=peptide.display&ID=13)  peptide, Co (26 aa)

s-w opt: 40 Z-score: 80.8 bits: 14.8 E(): 8.4

Smith-Waterman score: 40; 63.6% identity (72.7% similar) in 11 aa overlap (2-12:8-18)

10

Pep4 DKIVKKIFKKYS

: ::.::: :

#BIOFI GRFKRFRKKFKKLFKKLSPVIPLLHL

10 20

>>[BaAMP:14](http://www.baamps.it/browse?task=peptide.display&ID=14)  peptide, Co (26 aa)

s-w opt: 40 Z-score: 80.8 bits: 14.8 E(): 8.4

Smith-Waterman score: 40; 63.6% identity (72.7% similar) in 11 aa overlap (2-12:8-18)

10

Pep4 DKIVKKIFKKYS

: ::.::: :

#BIOFI GRFKRFRKKFKKLFKKLSPVIPLLHL

10 20

>>[BaAMP:14](http://www.baamps.it/browse?task=peptide.display&ID=14)  peptide, Co (26 aa)

s-w opt: 40 Z-score: 80.8 bits: 14.8 E(): 8.4

Smith-Waterman score: 40; 63.6% identity (72.7% similar) in 11 aa overlap (2-12:8-18)

10

Pep4 DKIVKKIFKKYS

: ::.::: :

#BIOFI GRFKRFRKKFKKLFKKLSPVIPLLHL

10 20

>>[BaAMP:150](http://www.baamps.it/browse?task=peptide.display&ID=150)  peptide, (26 aa)

s-w opt: 40 Z-score: 80.8 bits: 14.8 E(): 8.4

Smith-Waterman score: 40; 63.6% identity (72.7% similar) in 11 aa overlap (2-12:8-18)

10

Pep4 DKIVKKIFKKYS

: ::.::: :

#BIOFI GRFKRFRKKFKKLFKKLSPVIPLLHL

10 20

>>[BaAMP:159](http://www.baamps.it/browse?task=peptide.display&ID=159)  peptide, (26 aa)

s-w opt: 40 Z-score: 80.8 bits: 14.8 E(): 8.4

Smith-Waterman score: 40; 63.6% identity (72.7% similar) in 11 aa overlap (2-12:8-18)

10

Pep4 DKIVKKIFKKYS

: ::.::: :

#BIOFI GRFKRFRKKFKKLFKKLSPVIPLLHL

10 20

>>[BaAMP:159](http://www.baamps.it/browse?task=peptide.display&ID=159)  peptide, (26 aa)

s-w opt: 40 Z-score: 80.8 bits: 14.8 E(): 8.4

Smith-Waterman score: 40; 63.6% identity (72.7% similar) in 11 aa overlap (2-12:8-18)

10

Pep4 DKIVKKIFKKYS

: ::.::: :

#BIOFI GRFKRFRKKFKKLFKKLSPVIPLLHL

10 20

>>[BaAMP:15](http://www.baamps.it/browse?task=peptide.display&ID=15)  peptide, Co (26 aa)

s-w opt: 40 Z-score: 80.8 bits: 14.8 E(): 8.4

Smith-Waterman score: 40; 63.6% identity (72.7% similar) in 11 aa overlap (2-12:8-18)

10

Pep4 DKIVKKIFKKYS

: ::.::: :

#BIOFI GRFKRFRKKFKKLFKKLSPVIPLLHL

10 20

>>[BaAMP:15](http://www.baamps.it/browse?task=peptide.display&ID=15)  peptide, Co (26 aa)

s-w opt: 40 Z-score: 80.8 bits: 14.8 E(): 8.4

Smith-Waterman score: 40; 63.6% identity (72.7% similar) in 11 aa overlap (2-12:8-18)

10

Pep4 DKIVKKIFKKYS

: ::.::: :

#BIOFI GRFKRFRKKFKKLFKKLSPVIPLLHL

10 20

>>[BaAMP:160](http://www.baamps.it/browse?task=peptide.display&ID=160)  peptide, (26 aa)

s-w opt: 40 Z-score: 80.8 bits: 14.8 E(): 8.4

Smith-Waterman score: 40; 63.6% identity (72.7% similar) in 11 aa overlap (2-12:8-18)

10

Pep4 DKIVKKIFKKYS

: ::.::: :

#BIOFI GRFKRFRKKFKKLFKKLSPVIPLLHL

10 20

>>[BaAMP:160](http://www.baamps.it/browse?task=peptide.display&ID=160)  peptide, (26 aa)

s-w opt: 40 Z-score: 80.8 bits: 14.8 E(): 8.4

Smith-Waterman score: 40; 63.6% identity (72.7% similar) in 11 aa overlap (2-12:8-18)

10

Pep4 DKIVKKIFKKYS

: ::.::: :

#BIOFI GRFKRFRKKFKKLFKKLSPVIPLLHL

10 20

>>[BaAMP:4](http://www.baamps.it/browse?task=peptide.display&ID=4)  peptide, Conc (26 aa)

s-w opt: 40 Z-score: 80.8 bits: 14.8 E(): 8.4

Smith-Waterman score: 40; 63.6% identity (72.7% similar) in 11 aa overlap (2-12:8-18)

10

Pep4 DKIVKKIFKKYS

: ::.::: :

#BIOFI GRFKRFRKKFKKLFKKLSPVIPLLHL

10 20

>>[BaAMP:7](http://www.baamps.it/browse?task=peptide.display&ID=7)  peptide, Conc (26 aa)

s-w opt: 40 Z-score: 80.8 bits: 14.8 E(): 8.4

Smith-Waterman score: 40; 63.6% identity (72.7% similar) in 11 aa overlap (2-12:8-18)

10

Pep4 DKIVKKIFKKYS

: ::.::: :

#BIOFI GRFKRFRKKFKKLFKKLSPVIPLLHL

10 20

>>[BaAMP:215](http://www.baamps.it/browse?task=peptide.display&ID=215)  peptide, (20 aa)

s-w opt: 38 Z-score: 79.8 bits: 14.2 E(): 9.7

Smith-Waterman score: 38; 50.0% identity (90.0% similar) in 10 aa overlap (3-12:6-15)

10

Pep4 DKIVKKIFKKYS

..::..: ::

#BIOFI ALWKTLLKKVLKAYSPWTNF

10 20

>>[BaAMP:216](http://www.baamps.it/browse?task=peptide.display&ID=216)  peptide, (20 aa)

s-w opt: 38 Z-score: 79.8 bits: 14.2 E(): 9.7

Smith-Waterman score: 38; 50.0% identity (90.0% similar) in 10 aa overlap (3-12:6-15)

10

Pep4 DKIVKKIFKKYS

..::..: ::

#BIOFI ALWKTLLKKVLKAYSPWTNF

10 20

5>>>Pep5 - 12 aa

Library: BIOFIN/ssearch-baamp/biofin 15265 residues in 786 sequences

opt E()

< 20 0 0:

22 0 0: one = represents 2 library sequences

24 0 0:

26 1 0:=

28 0 0:

30 0 1:*

32 0 4: *

34 34 11:=====*===========

36 26 23:===========*=

38 67 39:===================*==============

40 39 54:==================== *

42 49 66:========================= *

44 43 72:====================== *

46 52 74:========================== *

48 60 71:============================== *

50 69 65:================================*==

52 47 57:======================== *

54 78 48:=======================*===============

56 56 40:===================*========

58 81 33:================*========================

60 25 27:=============*

62 19 22:==========*

64 12 17:====== *

66 4 14:== *

68 6 11:=== *

70 5 8:===*

72 0 7: *

74 2 5:= *

76 11 4:=*====

78 0 3: *

80 0 2:*

82 0 2:*

84 0 1:*

86 0 1:*

88 0 1:*

90 0 1:*

92 0 1:*

94 0 0:

96 0 0:

98 0 0:

100 0 0:

102 0 0:

104 0 0:

106 0 0:

108 0 0:

110 0 0:

112 0 0:

114 0 0:

116 0 0:

118 0 0:

>120 0 0:

15265 residues in 786 sequences

Statistics: MLE_cen statistics: Lambda= 0.2168; K= 0.123 (cen=39)

Kolmogorov-Smirnov statistic: 0.0628 (N=21) at 52

Algorithm: Smith-Waterman (SSE2, Michael Farrar 2006) (6.0 Mar 2007)

Parameters: BL50 matrix (15:-5), open/ext: -10/-2

Scan time: 0.000

!! No sequences with E() < 10.000000

6>>>Pep6 - 12 aa

Library: BIOFIN/ssearch-baamp/biofin 15265 residues in 786 sequences

opt E()

< 20 0 0:

22 0 0: one = represents 3 library sequences

24 0 0:

26 0 0:

28 0 0:

30 0 1:*

32 9 4:=*=

34 23 11:===*====

36 54 23:=======*==========

38 27 39:========= *

40 26 54:========= *

42 64 66:=====================*

44 32 72:=========== *

46 94 74:========================*=======

48 52 71:================== *

50 148 65:=====================*============================

52 52 57:==================*

54 48 48:===============*

56 29 40:========== *

58 10 33:==== *

60 18 27:====== *

62 14 22:===== *

64 24 17:=====*==

66 13 14:====*

68 6 11:== *

70 0 8: *

72 4 7:==*

74 16 5:=*====

76 2 4:=*

78 21 3:*======

80 0 2:*

82 0 2:*

84 0 1:*

86 0 1:*

88 0 1:* inset = represents 1 library sequences

90 0 1:*

92 0 1:* :*

94 0 0: *

96 0 0: *

98 0 0: *

100 0 0: *

102 0 0: *

104 0 0: *

106 0 0: *

108 0 0: *

110 0 0: *

112 0 0: *

114 0 0: *

116 0 0: *

118 0 0: *

>120 0 0: *

15265 residues in 786 sequences

Statistics: MLE_cen statistics: Lambda= 0.1968; K=0.09747 (cen=39)

Kolmogorov-Smirnov statistic: 0.0628 (N=23) at 50

Algorithm: Smith-Waterman (SSE2, Michael Farrar 2006) (6.0 Mar 2007)

Parameters: BL50 matrix (15:-5), open/ext: -10/-2

Scan time: 0.000

!! No sequences with E() < 10.000000

7>>>Pep7 - 12 aa

Library: BIOFIN/ssearch-baamp/biofin 15265 residues in 786 sequences

opt E()

< 20 0 0:

22 0 0: one = represents 2 library sequences

24 0 0:

26 0 0:

28 1 0:=

30 1 1:*

32 1 4:=*

34 31 11:=====*==========

36 25 23:===========*=

38 28 39:============== *

40 64 54:==========================*=====

42 54 66:=========================== *

44 91 72:===================================*==========

46 73 74:====================================*

48 49 71:========================= *

50 69 65:================================*==

52 64 57:============================*===

54 31 48:================ *

56 7 40:==== *

58 20 33:========== *

60 93 27:=============*=================================

62 17 22:========= *

64 11 17:====== *

66 0 14: *

68 23 11:=====*======

70 4 8:== *

72 0 7: *

74 1 5:= *

76 17 4:=*=======

78 7 3:=*==

80 4 2:*=

82 0 2:*

84 0 1:*

86 0 1:*

88 0 1:*

90 0 1:*

92 0 1:*

94 0 0:

96 0 0:

98 0 0:

100 0 0:

102 0 0:

104 0 0:

106 0 0:

108 0 0:

110 0 0:

112 0 0:

114 0 0:

116 0 0:

118 0 0:

>120 0 0:

15265 residues in 786 sequences

Statistics: MLE_cen statistics: Lambda= 0.2246; K=0.1529 (cen=39)

Kolmogorov-Smirnov statistic: 0.0630 (N=23) at 58

Algorithm: Smith-Waterman (SSE2, Michael Farrar 2006) (6.0 Mar 2007)

Parameters: BL50 matrix (15:-5), open/ext: -10/-2

Scan time: 0.000

The best scores are: s-w bits E(786)

[BaAMP:80](http://www.baamps.it/browse?task=peptide.display&ID=80)  peptide ( 20) 36 14.4 8.8

[BaAMP:83](http://www.baamps.it/browse?task=peptide.display&ID=83)  peptide ( 20) 36 14.4 8.8

[BaAMP:83](http://www.baamps.it/browse?task=peptide.display&ID=83)  peptide ( 20) 36 14.4 8.8

[BaAMP:80](http://www.baamps.it/browse?task=peptide.display&ID=80)  peptide ( 20) 36 14.4 8.8

[BaAMP:358](http://www.baamps.it/browse?task=peptide.display&ID=358)  pepti ( 22) 36 14.4 9.7

[BaAMP:359](http://www.baamps.it/browse?task=peptide.display&ID=359)  pepti ( 22) 36 14.4 9.7

>>[BaAMP:80](http://www.baamps.it/browse?task=peptide.display&ID=80)  peptide, Co (20 aa)

s-w opt: 36 Z-score: 80.5 bits: 14.4 E(): 8.8

Smith-Waterman score: 36; 45.5% identity (81.8% similar) in 11 aa overlap (1-11:8-18)

10

Pep7 IKKAYRKLSKKY

:.:.:. : :.

#BIOFI LAHQKPFIRKSYKCLHKRCR

10 20

>>[BaAMP:83](http://www.baamps.it/browse?task=peptide.display&ID=83)  peptide, Co (20 aa)

s-w opt: 36 Z-score: 80.5 bits: 14.4 E(): 8.8

Smith-Waterman score: 36; 45.5% identity (81.8% similar) in 11 aa overlap (1-11:8-18)

10

Pep7 IKKAYRKLSKKY

:.:.:. : :.

#BIOFI LAHQKPFIRKSYKCLHKRCR

10 20

>>[BaAMP:83](http://www.baamps.it/browse?task=peptide.display&ID=83)  peptide, Co (20 aa)

s-w opt: 36 Z-score: 80.5 bits: 14.4 E(): 8.8

Smith-Waterman score: 36; 45.5% identity (81.8% similar) in 11 aa overlap (1-11:8-18)

10

Pep7 IKKAYRKLSKKY

:.:.:. : :.

#BIOFI LAHQKPFIRKSYKCLHKRCR

10 20

>>[BaAMP:80](http://www.baamps.it/browse?task=peptide.display&ID=80)  peptide, Co (20 aa)

s-w opt: 36 Z-score: 80.5 bits: 14.4 E(): 8.8

Smith-Waterman score: 36; 45.5% identity (81.8% similar) in 11 aa overlap (1-11:8-18)

10

Pep7 IKKAYRKLSKKY

:.:.:. : :.

#BIOFI LAHQKPFIRKSYKCLHKRCR

10 20

>>[BaAMP:358](http://www.baamps.it/browse?task=peptide.display&ID=358)  peptide, (22 aa)

s-w opt: 36 Z-score: 79.7 bits: 14.4 E(): 9.7

Smith-Waterman score: 36; 63.6% identity (72.7% similar) in 11 aa overlap (1-11:7-17)

10

Pep7 IKKAYRKLSKKY

::: .:: ::

#BIOFI GIWKKWIKKWLKKLLKKLWKKG

10 20

>>[BaAMP:359](http://www.baamps.it/browse?task=peptide.display&ID=359)  peptide, (22 aa)

s-w opt: 36 Z-score: 79.7 bits: 14.4 E(): 9.7

Smith-Waterman score: 36; 63.6% identity (72.7% similar) in 11 aa overlap (1-11:7-17)

10

Pep7 IKKAYRKLSKKY

::: .:: ::

#BIOFI GIWKKWIKKWLKKLLKKLWKKG

10 20

8>>>Pep8 - 12 aa

Library: BIOFIN/ssearch-baamp/biofin 15265 residues in 786 sequences

opt E()

< 20 0 0:

22 0 0: one = represents 2 library sequences

24 0 0:

26 0 0:

28 0 0:

30 1 1:*

32 36 4:=*================

34 7 11:==== *

36 33 23:===========*=====

38 10 39:===== *

40 38 54:=================== *

42 49 66:========================= *

44 54 72:=========================== *

46 60 74:============================== *

48 103 71:===================================*================

50 98 65:================================*================

52 76 57:============================*=========

54 65 48:=======================*=========

56 12 40:====== *

58 8 33:==== *

60 61 27:=============*=================

62 6 22:=== *

64 6 17:=== *

66 1 14:= *

68 7 11:==== *

70 1 8:= *

72 4 7:== *

74 15 5:==*=====

76 16 4:=*======

78 0 3: *

80 4 2:*=

82 15 2:*=======

84 0 1:*

86 0 1:*

88 0 1:*

90 0 1:*

92 0 1:*

94 0 0:

96 0 0:

98 0 0:

100 0 0:

102 0 0:

104 0 0:

106 0 0:

108 0 0:

110 0 0:

112 0 0:

114 0 0:

116 0 0:

118 0 0:

>120 0 0:

15265 residues in 786 sequences

Statistics: MLE_cen statistics: Lambda= 0.1894; K=0.1277 (cen=39)

Kolmogorov-Smirnov statistic: 0.0719 (N=25) at 46

Algorithm: Smith-Waterman (SSE2, Michael Farrar 2006) (6.0 Mar 2007)

Parameters: BL50 matrix (15:-5), open/ext: -10/-2

Scan time: 0.000

The best scores are: s-w bits E(786)

[BaAMP:537](http://www.baamps.it/browse?task=peptide.display&ID=537)  pepti ( 11) 40 13.9 6.8

[BaAMP:540](http://www.baamps.it/browse?task=peptide.display&ID=540)  pepti ( 11) 40 13.9 6.8

[BaAMP:536](http://www.baamps.it/browse?task=peptide.display&ID=536)  pepti ( 11) 40 13.9 6.8

[BaAMP:538](http://www.baamps.it/browse?task=peptide.display&ID=538)  pepti ( 11) 40 13.9 6.8

[BaAMP:542](http://www.baamps.it/browse?task=peptide.display&ID=542)  pepti ( 11) 40 13.9 6.8

[BaAMP:537](http://www.baamps.it/browse?task=peptide.display&ID=537)  pepti ( 11) 40 13.9 6.8

[BaAMP:159](http://www.baamps.it/browse?task=peptide.display&ID=159)  pepti ( 26) 44 15.0 7.5

[BaAMP:13](http://www.baamps.it/browse?task=peptide.display&ID=13)  peptide ( 26) 44 15.0 7.5

[BaAMP:13](http://www.baamps.it/browse?task=peptide.display&ID=13)  peptide ( 26) 44 15.0 7.5

[BaAMP:160](http://www.baamps.it/browse?task=peptide.display&ID=160)  pepti ( 26) 44 15.0 7.5

[BaAMP:160](http://www.baamps.it/browse?task=peptide.display&ID=160)  pepti ( 26) 44 15.0 7.5

[BaAMP:4](http://www.baamps.it/browse?task=peptide.display&ID=4)  peptide, ( 26) 44 15.0 7.5

[BaAMP:7](http://www.baamps.it/browse?task=peptide.display&ID=7)  peptide, ( 26) 44 15.0 7.5

[BaAMP:159](http://www.baamps.it/browse?task=peptide.display&ID=159)  pepti ( 26) 44 15.0 7.5

[BaAMP:15](http://www.baamps.it/browse?task=peptide.display&ID=15)  peptide ( 26) 44 15.0 7.5

[BaAMP:15](http://www.baamps.it/browse?task=peptide.display&ID=15)  peptide ( 26) 44 15.0 7.5

[BaAMP:14](http://www.baamps.it/browse?task=peptide.display&ID=14)  peptide ( 26) 44 15.0 7.5

[BaAMP:14](http://www.baamps.it/browse?task=peptide.display&ID=14)  peptide ( 26) 44 15.0 7.5

[BaAMP:150](http://www.baamps.it/browse?task=peptide.display&ID=150)  pepti ( 26) 44 15.0 7.5

>>[BaAMP:537](http://www.baamps.it/browse?task=peptide.display&ID=537)  peptide, (11 aa)

s-w opt: 40 Z-score: 82.6 bits: 13.9 E(): 6.8

Smith-Waterman score: 40; 66.7% identity (100.0% similar) in 9 aa overlap (2-10:1-9)

10

Pep8 IKQVKKLFKKWG

:..::.:::

#BIOFI KRAKKFFKKLK

10

>>[BaAMP:540](http://www.baamps.it/browse?task=peptide.display&ID=540)  peptide, (11 aa)

s-w opt: 40 Z-score: 82.6 bits: 13.9 E(): 6.8

Smith-Waterman score: 40; 66.7% identity (100.0% similar) in 9 aa overlap (2-10:1-9)

10

Pep8 IKQVKKLFKKWG

:..::.:::

#BIOFI KRAKKFFKKLK

10

>>[BaAMP:536](http://www.baamps.it/browse?task=peptide.display&ID=536)  peptide, (11 aa)

s-w opt: 40 Z-score: 82.6 bits: 13.9 E(): 6.8

Smith-Waterman score: 40; 66.7% identity (100.0% similar) in 9 aa overlap (2-10:1-9)

10

Pep8 IKQVKKLFKKWG

:..::.:::

#BIOFI KRAKKFFKKLK

10

>>[BaAMP:538](http://www.baamps.it/browse?task=peptide.display&ID=538)  peptide, (11 aa)

s-w opt: 40 Z-score: 82.6 bits: 13.9 E(): 6.8

Smith-Waterman score: 40; 66.7% identity (100.0% similar) in 9 aa overlap (2-10:1-9)

10

Pep8 IKQVKKLFKKWG

:..::.:::

#BIOFI KRAKKFFKKPK

10

>>[BaAMP:542](http://www.baamps.it/browse?task=peptide.display&ID=542)  peptide, (11 aa)

s-w opt: 40 Z-score: 82.6 bits: 13.9 E(): 6.8

Smith-Waterman score: 40; 66.7% identity (100.0% similar) in 9 aa overlap (2-10:1-9)

10

Pep8 IKQVKKLFKKWG

:..::.:::

#BIOFI KRAKKFFKKPK

10

>>[BaAMP:537](http://www.baamps.it/browse?task=peptide.display&ID=537)  peptide, (11 aa)

s-w opt: 40 Z-score: 82.6 bits: 13.9 E(): 6.8

Smith-Waterman score: 40; 66.7% identity (100.0% similar) in 9 aa overlap (2-10:1-9)

10

Pep8 IKQVKKLFKKWG

:..::.:::

#BIOFI KRAKKFFKKLK

10

>>[BaAMP:159](http://www.baamps.it/browse?task=peptide.display&ID=159)  peptide, (26 aa)

s-w opt: 44 Z-score: 81.8 bits: 15.0 E(): 7.5

Smith-Waterman score: 44; 77.8% identity (88.9% similar) in 9 aa overlap (2-10:8-16)

10

Pep8 IKQVKKLFKKWG

:. ::::::

#BIOFI GRFKRFRKKFKKLFKKLSPVIPLLHL

10 20

>>[BaAMP:13](http://www.baamps.it/browse?task=peptide.display&ID=13)  peptide, Co (26 aa)

s-w opt: 44 Z-score: 81.8 bits: 15.0 E(): 7.5

Smith-Waterman score: 44; 77.8% identity (88.9% similar) in 9 aa overlap (2-10:8-16)

10

Pep8 IKQVKKLFKKWG

:. ::::::

#BIOFI GRFKRFRKKFKKLFKKLSPVIPLLHL

10 20

>>[BaAMP:13](http://www.baamps.it/browse?task=peptide.display&ID=13)  peptide, Co (26 aa)

s-w opt: 44 Z-score: 81.8 bits: 15.0 E(): 7.5

Smith-Waterman score: 44; 77.8% identity (88.9% similar) in 9 aa overlap (2-10:8-16)

10

Pep8 IKQVKKLFKKWG

:. ::::::

#BIOFI GRFKRFRKKFKKLFKKLSPVIPLLHL

10 20

>>[BaAMP:160](http://www.baamps.it/browse?task=peptide.display&ID=160)  peptide, (26 aa)

s-w opt: 44 Z-score: 81.8 bits: 15.0 E(): 7.5

Smith-Waterman score: 44; 77.8% identity (88.9% similar) in 9 aa overlap (2-10:8-16)

10

Pep8 IKQVKKLFKKWG

:. ::::::

#BIOFI GRFKRFRKKFKKLFKKLSPVIPLLHL

10 20

>>[BaAMP:160](http://www.baamps.it/browse?task=peptide.display&ID=160)  peptide, (26 aa)

s-w opt: 44 Z-score: 81.8 bits: 15.0 E(): 7.5

Smith-Waterman score: 44; 77.8% identity (88.9% similar) in 9 aa overlap (2-10:8-16)

10

Pep8 IKQVKKLFKKWG

:. ::::::

#BIOFI GRFKRFRKKFKKLFKKLSPVIPLLHL

10 20

>>[BaAMP:4](http://www.baamps.it/browse?task=peptide.display&ID=4)  peptide, Conc (26 aa)

s-w opt: 44 Z-score: 81.8 bits: 15.0 E(): 7.5

Smith-Waterman score: 44; 77.8% identity (88.9% similar) in 9 aa overlap (2-10:8-16)

10

Pep8 IKQVKKLFKKWG

:. ::::::

#BIOFI GRFKRFRKKFKKLFKKLSPVIPLLHL

10 20

>>[BaAMP:7](http://www.baamps.it/browse?task=peptide.display&ID=7)  peptide, Conc (26 aa)

s-w opt: 44 Z-score: 81.8 bits: 15.0 E(): 7.5

Smith-Waterman score: 44; 77.8% identity (88.9% similar) in 9 aa overlap (2-10:8-16)

10

Pep8 IKQVKKLFKKWG

:. ::::::

#BIOFI GRFKRFRKKFKKLFKKLSPVIPLLHL

10 20

>>[BaAMP:159](http://www.baamps.it/browse?task=peptide.display&ID=159)  peptide, (26 aa)

s-w opt: 44 Z-score: 81.8 bits: 15.0 E(): 7.5

Smith-Waterman score: 44; 77.8% identity (88.9% similar) in 9 aa overlap (2-10:8-16)

10

Pep8 IKQVKKLFKKWG

:. ::::::

#BIOFI GRFKRFRKKFKKLFKKLSPVIPLLHL

10 20

>>[BaAMP:15](http://www.baamps.it/browse?task=peptide.display&ID=15)  peptide, Co (26 aa)

s-w opt: 44 Z-score: 81.8 bits: 15.0 E(): 7.5

Smith-Waterman score: 44; 77.8% identity (88.9% similar) in 9 aa overlap (2-10:8-16)

10

Pep8 IKQVKKLFKKWG

:. ::::::

#BIOFI GRFKRFRKKFKKLFKKLSPVIPLLHL

10 20

>>[BaAMP:15](http://www.baamps.it/browse?task=peptide.display&ID=15)  peptide, Co (26 aa)

s-w opt: 44 Z-score: 81.8 bits: 15.0 E(): 7.5

Smith-Waterman score: 44; 77.8% identity (88.9% similar) in 9 aa overlap (2-10:8-16)

10

Pep8 IKQVKKLFKKWG

:. ::::::

#BIOFI GRFKRFRKKFKKLFKKLSPVIPLLHL

10 20

>>[BaAMP:14](http://www.baamps.it/browse?task=peptide.display&ID=14)  peptide, Co (26 aa)

s-w opt: 44 Z-score: 81.8 bits: 15.0 E(): 7.5

Smith-Waterman score: 44; 77.8% identity (88.9% similar) in 9 aa overlap (2-10:8-16)

10

Pep8 IKQVKKLFKKWG

:. ::::::

#BIOFI GRFKRFRKKFKKLFKKLSPVIPLLHL

10 20

>>[BaAMP:14](http://www.baamps.it/browse?task=peptide.display&ID=14)  peptide, Co (26 aa)

s-w opt: 44 Z-score: 81.8 bits: 15.0 E(): 7.5

Smith-Waterman score: 44; 77.8% identity (88.9% similar) in 9 aa overlap (2-10:8-16)

10

Pep8 IKQVKKLFKKWG

:. ::::::

#BIOFI GRFKRFRKKFKKLFKKLSPVIPLLHL

10 20

>>[BaAMP:150](http://www.baamps.it/browse?task=peptide.display&ID=150)  peptide, (26 aa)

s-w opt: 44 Z-score: 81.8 bits: 15.0 E(): 7.5

Smith-Waterman score: 44; 77.8% identity (88.9% similar) in 9 aa overlap (2-10:8-16)

10

Pep8 IKQVKKLFKKWG

:. ::::::

#BIOFI GRFKRFRKKFKKLFKKLSPVIPLLHL

10 20

9>>>Pep9 - 12 aa

Library: BIOFIN/ssearch-baamp/biofin 15265 residues in 786 sequences

opt E()

< 20 0 0:

22 0 0: one = represents 3 library sequences

24 0 0:

26 0 0:

28 0 0:

30 0 1:*

32 9 4:=*=

34 22 11:===*====

36 32 23:=======*===

38 25 39:========= *

40 50 54:=================*

42 59 66:==================== *

44 53 72:================== *

46 85 74:========================*====

48 54 71:================== *

50 148 65:=====================*============================

52 41 57:============== *

54 57 48:===============*===

56 27 40:========= *

58 9 33:=== *

60 15 27:===== *

62 17 22:====== *

64 23 17:=====*==

66 10 14:====*

68 7 11:===*

70 1 8:= *

72 2 7:= *

74 2 5:=*

76 8 4:=*=

78 25 3:*========

80 5 2:*=

82 0 2:*

84 0 1:*

86 0 1:*

88 0 1:* inset = represents 1 library sequences

90 0 1:*

92 0 1:* :*

94 0 0: *

96 0 0: *

98 0 0: *

100 0 0: *

102 0 0: *

104 0 0: *

106 0 0: *

108 0 0: *

110 0 0: *

112 0 0: *

114 0 0: *

116 0 0: *

118 0 0: *

>120 0 0: *

15265 residues in 786 sequences

Statistics: MLE_cen statistics: Lambda= 0.1942; K=0.09491 (cen=39)

Kolmogorov-Smirnov statistic: 0.0729 (N=25) at 50

Algorithm: Smith-Waterman (SSE2, Michael Farrar 2006) (6.0 Mar 2007)

Parameters: BL50 matrix (15:-5), open/ext: -10/-2

Scan time: 0.000

The best scores are: s-w bits E(786)

[BaAMP:13](http://www.baamps.it/browse?task=peptide.display&ID=13)  peptide ( 26) 40 14.6 9.8

[BaAMP:13](http://www.baamps.it/browse?task=peptide.display&ID=13)  peptide ( 26) 40 14.6 9.8

[BaAMP:14](http://www.baamps.it/browse?task=peptide.display&ID=14)  peptide ( 26) 40 14.6 9.8

[BaAMP:14](http://www.baamps.it/browse?task=peptide.display&ID=14)  peptide ( 26) 40 14.6 9.8

[BaAMP:150](http://www.baamps.it/browse?task=peptide.display&ID=150)  pepti ( 26) 40 14.6 9.8

[BaAMP:159](http://www.baamps.it/browse?task=peptide.display&ID=159)  pepti ( 26) 40 14.6 9.8

[BaAMP:159](http://www.baamps.it/browse?task=peptide.display&ID=159)  pepti ( 26) 40 14.6 9.8

[BaAMP:15](http://www.baamps.it/browse?task=peptide.display&ID=15)  peptide ( 26) 40 14.6 9.8

[BaAMP:15](http://www.baamps.it/browse?task=peptide.display&ID=15)  peptide ( 26) 40 14.6 9.8

[BaAMP:160](http://www.baamps.it/browse?task=peptide.display&ID=160)  pepti ( 26) 40 14.6 9.8

[BaAMP:160](http://www.baamps.it/browse?task=peptide.display&ID=160)  pepti ( 26) 40 14.6 9.8

[BaAMP:4](http://www.baamps.it/browse?task=peptide.display&ID=4)  peptide, ( 26) 40 14.6 9.8

[BaAMP:7](http://www.baamps.it/browse?task=peptide.display&ID=7)  peptide, ( 26) 40 14.6 9.8

>>[BaAMP:13](http://www.baamps.it/browse?task=peptide.display&ID=13)  peptide, Co (26 aa)

s-w opt: 40 Z-score: 79.6 bits: 14.6 E(): 9.8

Smith-Waterman score: 40; 63.6% identity (72.7% similar) in 11 aa overlap (1-11:8-18)

10

Pep9 KIVKKIFKKYSE

: ::.::: :

#BIOFI GRFKRFRKKFKKLFKKLSPVIPLLHL

10 20

>>[BaAMP:13](http://www.baamps.it/browse?task=peptide.display&ID=13)  peptide, Co (26 aa)

s-w opt: 40 Z-score: 79.6 bits: 14.6 E(): 9.8

Smith-Waterman score: 40; 63.6% identity (72.7% similar) in 11 aa overlap (1-11:8-18)

10

Pep9 KIVKKIFKKYSE

: ::.::: :

#BIOFI GRFKRFRKKFKKLFKKLSPVIPLLHL

10 20

>>[BaAMP:14](http://www.baamps.it/browse?task=peptide.display&ID=14)  peptide, Co (26 aa)

s-w opt: 40 Z-score: 79.6 bits: 14.6 E(): 9.8

Smith-Waterman score: 40; 63.6% identity (72.7% similar) in 11 aa overlap (1-11:8-18)

10

Pep9 KIVKKIFKKYSE

: ::.::: :

#BIOFI GRFKRFRKKFKKLFKKLSPVIPLLHL

10 20

>>[BaAMP:14](http://www.baamps.it/browse?task=peptide.display&ID=14)  peptide, Co (26 aa)

s-w opt: 40 Z-score: 79.6 bits: 14.6 E(): 9.8

Smith-Waterman score: 40; 63.6% identity (72.7% similar) in 11 aa overlap (1-11:8-18)

10

Pep9 KIVKKIFKKYSE

: ::.::: :

#BIOFI GRFKRFRKKFKKLFKKLSPVIPLLHL

10 20

>>[BaAMP:150](http://www.baamps.it/browse?task=peptide.display&ID=150)  peptide, (26 aa)

s-w opt: 40 Z-score: 79.6 bits: 14.6 E(): 9.8

Smith-Waterman score: 40; 63.6% identity (72.7% similar) in 11 aa overlap (1-11:8-18)

10

Pep9 KIVKKIFKKYSE

: ::.::: :

#BIOFI GRFKRFRKKFKKLFKKLSPVIPLLHL

10 20

>>[BaAMP:159](http://www.baamps.it/browse?task=peptide.display&ID=159)  peptide, (26 aa)

s-w opt: 40 Z-score: 79.6 bits: 14.6 E(): 9.8

Smith-Waterman score: 40; 63.6% identity (72.7% similar) in 11 aa overlap (1-11:8-18)

10

Pep9 KIVKKIFKKYSE

: ::.::: :

#BIOFI GRFKRFRKKFKKLFKKLSPVIPLLHL

10 20

>>[BaAMP:159](http://www.baamps.it/browse?task=peptide.display&ID=159)  peptide, (26 aa)

s-w opt: 40 Z-score: 79.6 bits: 14.6 E(): 9.8

Smith-Waterman score: 40; 63.6% identity (72.7% similar) in 11 aa overlap (1-11:8-18)

10

Pep9 KIVKKIFKKYSE

: ::.::: :

#BIOFI GRFKRFRKKFKKLFKKLSPVIPLLHL

10 20

>>[BaAMP:15](http://www.baamps.it/browse?task=peptide.display&ID=15)  peptide, Co (26 aa)

s-w opt: 40 Z-score: 79.6 bits: 14.6 E(): 9.8

Smith-Waterman score: 40; 63.6% identity (72.7% similar) in 11 aa overlap (1-11:8-18)

10

Pep9 KIVKKIFKKYSE

: ::.::: :

#BIOFI GRFKRFRKKFKKLFKKLSPVIPLLHL

10 20

>>[BaAMP:15](http://www.baamps.it/browse?task=peptide.display&ID=15)  peptide, Co (26 aa)

s-w opt: 40 Z-score: 79.6 bits: 14.6 E(): 9.8

Smith-Waterman score: 40; 63.6% identity (72.7% similar) in 11 aa overlap (1-11:8-18)

10

Pep9 KIVKKIFKKYSE

: ::.::: :

#BIOFI GRFKRFRKKFKKLFKKLSPVIPLLHL

10 20

>>[BaAMP:160](http://www.baamps.it/browse?task=peptide.display&ID=160)  peptide, (26 aa)

s-w opt: 40 Z-score: 79.6 bits: 14.6 E(): 9.8

Smith-Waterman score: 40; 63.6% identity (72.7% similar) in 11 aa overlap (1-11:8-18)

10

Pep9 KIVKKIFKKYSE

: ::.::: :

#BIOFI GRFKRFRKKFKKLFKKLSPVIPLLHL

10 20

>>[BaAMP:160](http://www.baamps.it/browse?task=peptide.display&ID=160)  peptide, (26 aa)

s-w opt: 40 Z-score: 79.6 bits: 14.6 E(): 9.8

Smith-Waterman score: 40; 63.6% identity (72.7% similar) in 11 aa overlap (1-11:8-18)

10

Pep9 KIVKKIFKKYSE

: ::.::: :

#BIOFI GRFKRFRKKFKKLFKKLSPVIPLLHL

10 20

>>[BaAMP:4](http://www.baamps.it/browse?task=peptide.display&ID=4)  peptide, Conc (26 aa)

s-w opt: 40 Z-score: 79.6 bits: 14.6 E(): 9.8

Smith-Waterman score: 40; 63.6% identity (72.7% similar) in 11 aa overlap (1-11:8-18)

10

Pep9 KIVKKIFKKYSE

: ::.::: :

#BIOFI GRFKRFRKKFKKLFKKLSPVIPLLHL

10 20

>>[BaAMP:7](http://www.baamps.it/browse?task=peptide.display&ID=7)  peptide, Conc (26 aa)

s-w opt: 40 Z-score: 79.6 bits: 14.6 E(): 9.8

Smith-Waterman score: 40; 63.6% identity (72.7% similar) in 11 aa overlap (1-11:8-18)

10

Pep9 KIVKKIFKKYSE

: ::.::: :

#BIOFI GRFKRFRKKFKKLFKKLSPVIPLLHL

10 20

10>>>Pep10 - 12 aa

Library: BIOFIN/ssearch-baamp/biofin 15265 residues in 786 sequences

opt E()

< 20 0 0:

22 0 0: one = represents 2 library sequences

24 0 0:

26 0 0:

28 0 0:

30 1 1:*

32 0 4: *

34 30 11:=====*=========

36 34 23:===========*=====

38 31 39:================ *

40 63 54:==========================*=====

42 86 66:================================*==========

44 41 72:===================== *

46 81 74:====================================*====

48 43 71:====================== *

50 86 65:================================*==========

52 71 57:============================*=======

54 25 48:============= *

56 10 40:===== *

58 17 33:========= *

60 45 27:=============*=========

62 19 22:==========*

64 58 17:========*====================

66 15 14:======*=

68 20 11:=====*====

70 5 8:===*

72 4 7:== *

74 1 5:= *

76 0 4: *

78 0 3: *

80 0 2:*

82 0 2:*

84 0 1:*

86 0 1:*

88 0 1:*

90 0 1:*

92 0 1:*

94 0 0:

96 0 0:

98 0 0:

100 0 0:

102 0 0:

104 0 0:

106 0 0:

108 0 0:

110 0 0:

112 0 0:

114 0 0:

116 0 0:

118 0 0:

>120 0 0:

15265 residues in 786 sequences

Statistics: MLE_cen statistics: Lambda= 0.1970; K= 0.122 (cen=39)

Kolmogorov-Smirnov statistic: 0.0596 (N=21) at 42

Algorithm: Smith-Waterman (SSE2, Michael Farrar 2006) (6.0 Mar 2007)

Parameters: BL50 matrix (15:-5), open/ext: -10/-2

Scan time: 0.000

!! No sequences with E() < 10.000000

11>>>Pep11 - 12 aa

Library: BIOFIN/ssearch-baamp/biofin 15265 residues in 786 sequences

opt E()

< 20 0 0:

22 0 0: one = represents 2 library sequences

24 0 0:

26 0 0:

28 1 0:=

30 4 1:*=

32 0 4: *

34 1 11:= *

36 42 23:===========*=========

38 44 39:===================*==

40 60 54:==========================*===

42 84 66:================================*=========

44 64 72:================================ *

46 108 74:====================================*=================

48 67 71:================================== *

50 47 65:======================== *

52 23 57:============ *

54 28 48:============== *

56 26 40:============= *

58 50 33:================*========

60 9 27:===== *

62 14 22:======= *

64 2 17:= *

66 3 14:== *

68 80 11:=====*==================================

70 13 8:===*===

72 9 7:===*=

74 7 5:==*=

76 0 4: *

78 0 3: *

80 0 2:*

82 0 2:*

84 0 1:*

86 0 1:*

88 0 1:*

90 0 1:*

92 0 1:*

94 0 0:

96 0 0:

98 0 0:

100 0 0:

102 0 0:

104 0 0:

106 0 0:

108 0 0:

110 0 0:

112 0 0:

114 0 0:

116 0 0:

118 0 0:

>120 0 0:

15265 residues in 786 sequences

Statistics: MLE_cen statistics: Lambda= 0.2020; K=0.1109 (cen=39)

Kolmogorov-Smirnov statistic: 0.0808 (N=21) at 46

Algorithm: Smith-Waterman (SSE2, Michael Farrar 2006) (6.0 Mar 2007)

Parameters: BL50 matrix (15:-5), open/ext: -10/-2

Scan time: 0.000

!! No sequences with E() < 10.000000

12>>>Pep12 - 12 aa

Library: BIOFIN/ssearch-baamp/biofin 15265 residues in 786 sequences

opt E()

< 20 0 0:

22 0 0: one = represents 2 library sequences

24 0 0:

26 0 0:

28 0 0:

30 0 1:*

32 29 4:=*=============

34 15 11:=====*==

36 31 23:===========*====

38 21 39:=========== *

40 39 54:==================== *

42 38 66:=================== *

44 65 72:================================= *

46 58 74:============================= *

48 104 71:===================================*================

50 111 65:================================*=======================

52 63 57:============================*===

54 29 48:=============== *

56 7 40:==== *

58 40 33:================*===

60 12 27:====== *

62 18 22:========= *

64 12 17:====== *

66 1 14:= *

68 16 11:=====*==

70 40 8:===*================

72 12 7:===*==

74 4 5:==*

76 0 4: *

78 15 3:=*======

80 4 2:*=

82 0 2:*

84 2 1:*

86 0 1:*

88 0 1:*

90 0 1:*

92 0 1:*

94 0 0:

96 0 0:

98 0 0:

100 0 0:

102 0 0:

104 0 0:

106 0 0:

108 0 0:

110 0 0:

112 0 0:

114 0 0:

116 0 0:

118 0 0:

>120 0 0:

15265 residues in 786 sequences

Statistics: MLE_cen statistics: Lambda= 0.1754; K= 0.103 (cen=39)

Kolmogorov-Smirnov statistic: 0.0617 (N=25) at 46

Algorithm: Smith-Waterman (SSE2, Michael Farrar 2006) (6.0 Mar 2007)

Parameters: BL50 matrix (15:-5), open/ext: -10/-2

Scan time: 0.000

The best scores are: s-w bits E(786)

[BaAMP:560](http://www.baamps.it/browse?task=peptide.display&ID=560)  pepti ( 12) 44 14.4 5.2

[BaAMP:561](http://www.baamps.it/browse?task=peptide.display&ID=561)  pepti ( 12) 44 14.4 5.2

[BaAMP:536](http://www.baamps.it/browse?task=peptide.display&ID=536)  pepti ( 11) 40 13.4 9.5

[BaAMP:537](http://www.baamps.it/browse?task=peptide.display&ID=537)  pepti ( 11) 40 13.4 9.5

[BaAMP:540](http://www.baamps.it/browse?task=peptide.display&ID=540)  pepti ( 11) 40 13.4 9.5

[BaAMP:542](http://www.baamps.it/browse?task=peptide.display&ID=542)  pepti ( 11) 40 13.4 9.5

[BaAMP:537](http://www.baamps.it/browse?task=peptide.display&ID=537)  pepti ( 11) 40 13.4 9.5

[BaAMP:538](http://www.baamps.it/browse?task=peptide.display&ID=538)  pepti ( 11) 40 13.4 9.5

>>[BaAMP:560](http://www.baamps.it/browse?task=peptide.display&ID=560)  peptide, (12 aa)

s-w opt: 44 Z-score: 84.7 bits: 14.4 E(): 5.2

Smith-Waterman score: 44; 36.4% identity (72.7% similar) in 11 aa overlap (2-12:1-11)

10

Pep12 KQVKKLFKKWGW

..: ..: : :

#BIOFI KIKWILKYWKWS

10

>>[BaAMP:561](http://www.baamps.it/browse?task=peptide.display&ID=561)  peptide, (12 aa)

s-w opt: 44 Z-score: 84.7 bits: 14.4 E(): 5.2

Smith-Waterman score: 44; 36.4% identity (72.7% similar) in 11 aa overlap (2-12:1-11)

10

Pep12 KQVKKLFKKWGW

..: ..: : :

#BIOFI KIKWILKYWKWS

10

>>[BaAMP:536](http://www.baamps.it/browse?task=peptide.display&ID=536)  peptide, (11 aa)

s-w opt: 40 Z-score: 79.9 bits: 13.4 E(): 9.5

Smith-Waterman score: 40; 66.7% identity (100.0% similar) in 9 aa overlap (1-9:1-9)

10

Pep12 KQVKKLFKKWGW

:..::.:::

#BIOFI KRAKKFFKKLK

10

>>[BaAMP:537](http://www.baamps.it/browse?task=peptide.display&ID=537)  peptide, (11 aa)

s-w opt: 40 Z-score: 79.9 bits: 13.4 E(): 9.5

Smith-Waterman score: 40; 66.7% identity (100.0% similar) in 9 aa overlap (1-9:1-9)

10

Pep12 KQVKKLFKKWGW

:..::.:::

#BIOFI KRAKKFFKKLK

10

>>[BaAMP:540](http://www.baamps.it/browse?task=peptide.display&ID=540)  peptide, (11 aa)

s-w opt: 40 Z-score: 79.9 bits: 13.4 E(): 9.5

Smith-Waterman score: 40; 66.7% identity (100.0% similar) in 9 aa overlap (1-9:1-9)

10

Pep12 KQVKKLFKKWGW

:..::.:::

#BIOFI KRAKKFFKKLK

10

>>[BaAMP:542](http://www.baamps.it/browse?task=peptide.display&ID=542)  peptide, (11 aa)

s-w opt: 40 Z-score: 79.9 bits: 13.4 E(): 9.5

Smith-Waterman score: 40; 66.7% identity (100.0% similar) in 9 aa overlap (1-9:1-9)

10

Pep12 KQVKKLFKKWGW

:..::.:::

#BIOFI KRAKKFFKKPK

10

>>[BaAMP:537](http://www.baamps.it/browse?task=peptide.display&ID=537)  peptide, (11 aa)

s-w opt: 40 Z-score: 79.9 bits: 13.4 E(): 9.5

Smith-Waterman score: 40; 66.7% identity (100.0% similar) in 9 aa overlap (1-9:1-9)

10

Pep12 KQVKKLFKKWGW

:..::.:::

#BIOFI KRAKKFFKKLK

10

>>[BaAMP:538](http://www.baamps.it/browse?task=peptide.display&ID=538)  peptide, (11 aa)

s-w opt: 40 Z-score: 79.9 bits: 13.4 E(): 9.5

Smith-Waterman score: 40; 66.7% identity (100.0% similar) in 9 aa overlap (1-9:1-9)

10

Pep12 KQVKKLFKKWGW

:..::.:::

#BIOFI KRAKKFFKKPK

10

13>>>Pep13 - 12 aa

Library: BIOFIN/ssearch-baamp/biofin 15265 residues in 786 sequences

opt E()

< 20 0 0:

22 0 0: one = represents 3 library sequences

24 0 0:

26 0 0:

28 7 0:===

30 0 1:*

32 8 4:=*=

34 19 11:===*===

36 37 23:=======*=====

38 7 39:=== *

40 47 54:================ *

42 43 66:=============== *

44 21 72:======= *

46 108 74:========================*===========

48 96 71:=======================*========

50 107 65:=====================*==============

52 139 57:==================*============================

54 42 48:============== *

56 27 40:========= *

58 14 33:===== *

60 8 27:=== *

62 10 22:==== *

64 15 17:=====*

66 3 14:= *

68 2 11:= *

70 10 8:==*=

72 15 7:==*==

74 0 5: *

76 0 4: *

78 0 3:*

80 0 2:*

82 0 2:*

84 0 1:*

86 0 1:*

88 0 1:* inset = represents 1 library sequences

90 0 1:*

92 1 1:* :*

94 0 0: *

96 0 0: *

98 0 0: *

100 0 0: *

102 0 0: *

104 0 0: *

106 0 0: *

108 0 0: *

110 0 0: *

112 0 0: *

114 0 0: *

116 0 0: *

118 0 0: *

>120 0 0: *

15265 residues in 786 sequences

Statistics: MLE_cen statistics: Lambda= 0.2730; K=0.3041 (cen=39)

Kolmogorov-Smirnov statistic: 0.1306 (N=21) at 52

Algorithm: Smith-Waterman (SSE2, Michael Farrar 2006) (6.0 Mar 2007)

Parameters: BL50 matrix (15:-5), open/ext: -10/-2

Scan time: 0.000

The best scores are: s-w bits E(786)

[BaAMP:340](http://www.baamps.it/browse?task=peptide.display&ID=340)  pepti ( 12) 36 15.9 1.9

>>[BaAMP:340](http://www.baamps.it/browse?task=peptide.display&ID=340)  peptide, (12 aa)

s-w opt: 36 Z-score: 92.7 bits: 15.9 E(): 1.9

Smith-Waterman score: 36; 83.3% identity (100.0% similar) in 6 aa overlap (6-11:7-12)

10

Pep13 NRKKHVIRVCQD

:::::.

#BIOFI RLCRIVVIRVCR

10

14>>>Pep14 - 12 aa

Library: BIOFIN/ssearch-baamp/biofin 15265 residues in 786 sequences

opt E()

< 20 0 0:

22 0 0: one = represents 3 library sequences

24 0 0:

26 1 0:=

28 0 0:

30 0 1:*

32 9 4:=*=

34 28 11:===*======

36 2 23:= *

38 22 39:======== *

40 32 54:=========== *

42 93 66:=====================*=========

44 149 72:=======================*==========================

46 86 74:========================*====

48 44 71:=============== *

50 73 65:=====================*===

52 34 57:============ *

54 35 48:============ *

56 8 40:=== *

58 28 33:==========*

60 21 27:======= *

62 20 22:=======*

64 30 17:=====*====

66 6 14:== *

68 3 11:= *

70 5 8:==*

72 14 7:==*==

74 17 5:=*====

76 10 4:=*==

78 16 3:*=====

80 0 2:*

82 0 2:*

84 0 1:*

86 0 1:*

88 0 1:* inset = represents 1 library sequences

90 0 1:*

92 0 1:* :*

94 0 0: *

96 0 0: *

98 0 0: *

100 0 0: *

102 0 0: *

104 0 0: *

106 0 0: *

108 0 0: *

110 0 0: *

112 0 0: *

114 0 0: *

116 0 0: *

118 0 0: *

>120 0 0: *

15265 residues in 786 sequences

Statistics: MLE_cen statistics: Lambda= 0.2669; K=0.1197 (cen=39)

Kolmogorov-Smirnov statistic: 0.0986 (N=24) at 46

Algorithm: Smith-Waterman (SSE2, Michael Farrar 2006) (6.0 Mar 2007)

Parameters: BL50 matrix (15:-5), open/ext: -10/-2

Scan time: 0.000

!! No sequences with E() < 10.000000

15>>>Pep15 - 12 aa

Library: BIOFIN/ssearch-baamp/biofin 15265 residues in 786 sequences

opt E()

< 20 0 0:

22 0 0: one = represents 3 library sequences

24 0 0:

26 0 0:

28 0 0:

30 0 1:*

32 10 4:=*==

34 13 11:===*=

36 29 23:=======*==

38 19 39:======= *

40 64 54:=================*====

42 125 66:=====================*====================

44 48 72:================ *

46 94 74:========================*=======

48 77 71:=======================*==

50 49 65:================= *

52 23 57:======== *

54 12 48:==== *

56 42 40:=============*

58 27 33:========= *

60 5 27:== *

62 24 22:=======*

64 4 17:== *

66 68 14:====*==================

68 44 11:===*===========

70 3 8:= *

72 4 7:==*

74 0 5: *

76 2 4:=*

78 0 3:*

80 0 2:*

82 0 2:*

84 0 1:*

86 0 1:*

88 0 1:* inset = represents 1 library sequences

90 0 1:*

92 0 1:* :*

94 0 0: *

96 0 0: *

98 0 0: *

100 0 0: *

102 0 0: *

104 0 0: *

106 0 0: *

108 0 0: *

110 0 0: *

112 0 0: *

114 0 0: *

116 0 0: *

118 0 0: *

>120 0 0: *

15265 residues in 786 sequences

Statistics: MLE_cen statistics: Lambda= 0.2462; K=0.1731 (cen=39)

Kolmogorov-Smirnov statistic: 0.0812 (N=22) at 48

Algorithm: Smith-Waterman (SSE2, Michael Farrar 2006) (6.0 Mar 2007)

Parameters: BL50 matrix (15:-5), open/ext: -10/-2

Scan time: 0.000

!! No sequences with E() < 10.000000

16>>>Pep16 - 12 aa

Library: BIOFIN/ssearch-baamp/biofin 15265 residues in 786 sequences

opt E()

< 20 0 0:

22 0 0: one = represents 2 library sequences

24 0 0:

26 0 0:

28 0 0:

30 0 1:*

32 9 4:=*===

34 37 11:=====*=============

36 23 23:===========*

38 21 39:=========== *

40 34 54:================= *

42 77 66:================================*======

44 81 72:===================================*=====

46 51 74:========================== *

48 79 71:===================================*====

50 47 65:======================== *

52 46 57:======================= *

54 82 48:=======================*=================

56 60 40:===================*==========

58 12 33:====== *

60 6 27:=== *

62 41 22:==========*==========

64 20 17:========*=

66 19 14:======*===

68 7 11:==== *

70 26 8:===*=========

72 3 7:== *

74 5 5:==*

76 0 4: *

78 0 3: *

80 0 2:*

82 0 2:*

84 0 1:*

86 0 1:*

88 0 1:*

90 0 1:*

92 0 1:*

94 0 0:

96 0 0:

98 0 0:

100 0 0:

102 0 0:

104 0 0:

106 0 0:

108 0 0:

110 0 0:

112 0 0:

114 0 0:

116 0 0:

118 0 0:

>120 0 0:

15265 residues in 786 sequences

Statistics: MLE_cen statistics: Lambda= 0.2015; K=0.1026 (cen=39)

Kolmogorov-Smirnov statistic: 0.0399 (N=22) at 52

Algorithm: Smith-Waterman (SSE2, Michael Farrar 2006) (6.0 Mar 2007)

Parameters: BL50 matrix (15:-5), open/ext: -10/-2

Scan time: 0.010

!! No sequences with E() < 10.000000

17>>>Pep17 - 12 aa

Library: BIOFIN/ssearch-baamp/biofin 15265 residues in 786 sequences

opt E()

< 20 0 0:

22 0 0: one = represents 2 library sequences

24 0 0:

26 1 0:=

28 0 0:

30 0 1:*

32 0 4: *

34 41 11:=====*===============

36 44 23:===========*==========

38 42 39:===================*=

40 38 54:=================== *

42 37 66:=================== *

44 73 72:===================================*=

46 55 74:============================ *

48 119 71:===================================*========================

50 62 65:=============================== *

52 12 57:====== *

54 36 48:================== *

56 34 40:================= *

58 39 33:================*===

60 18 27:========= *

62 17 22:========= *

64 5 17:=== *

66 12 14:======*

68 65 11:=====*===========================

70 29 8:===*===========

72 3 7:== *

74 2 5:= *

76 0 4: *

78 2 3:=*

80 0 2:*

82 0 2:*

84 0 1:*

86 0 1:*

88 0 1:*

90 0 1:*

92 0 1:*

94 0 0:

96 0 0:

98 0 0:

100 0 0:

102 0 0:

104 0 0:

106 0 0:

108 0 0:

110 0 0:

112 0 0:

114 0 0:

116 0 0:

118 0 0:

>120 0 0:

15265 residues in 786 sequences

Statistics: MLE_cen statistics: Lambda= 0.2107; K=0.0781 (cen=39)

Kolmogorov-Smirnov statistic: 0.0670 (N=22) at 66

Algorithm: Smith-Waterman (SSE2, Michael Farrar 2006) (6.0 Mar 2007)

Parameters: BL50 matrix (15:-5), open/ext: -10/-2

Scan time: 0.000

!! No sequences with E() < 10.000000

18>>>Pep18 - 12 aa

Library: BIOFIN/ssearch-baamp/biofin 15265 residues in 786 sequences

opt E()

< 20 0 0:

22 0 0: one = represents 3 library sequences

24 1 0:=

26 0 0:

28 0 0:

30 0 1:*

32 0 4: *

34 21 11:===*===

36 48 23:=======*========

38 61 39:============*========

40 40 54:============== *

42 40 66:============== *

44 59 72:==================== *

46 34 74:============ *

48 52 71:================== *

50 40 65:============== *

52 142 57:==================*=============================

54 58 48:===============*====

56 67 40:=============*=========

58 40 33:==========*===

60 8 27:=== *

62 29 22:=======*==

64 7 17:=== *

66 12 14:====*

68 4 11:== *

70 6 8:==*

72 4 7:==*

74 0 5: *

76 0 4: *

78 13 3:*====

80 0 2:*

82 0 2:*

84 0 1:*

86 0 1:*

88 0 1:* inset = represents 1 library sequences

90 0 1:*

92 0 1:* :*

94 0 0: *

96 0 0: *

98 0 0: *

100 0 0: *

102 0 0: *

104 0 0: *

106 0 0: *

108 0 0: *

110 0 0: *

112 0 0: *

114 0 0: *

116 0 0: *

118 0 0: *

>120 0 0: *

15265 residues in 786 sequences

Statistics: MLE_cen statistics: Lambda= 0.2286; K=0.1329 (cen=39)

Kolmogorov-Smirnov statistic: 0.1064 (N=21) at 50

Algorithm: Smith-Waterman (SSE2, Michael Farrar 2006) (6.0 Mar 2007)

Parameters: BL50 matrix (15:-5), open/ext: -10/-2

Scan time: 0.000

!! No sequences with E() < 10.000000

19>>>Pep19 - 12 aa

Library: BIOFIN/ssearch-baamp/biofin 15265 residues in 786 sequences

opt E()

< 20 0 0:

22 0 0: one = represents 3 library sequences

24 1 0:=

26 7 0:===

28 0 0:

30 0 1:*

32 22 4:=*======

34 13 11:===*=

36 51 23:=======*=========

38 9 39:=== *

40 16 54:====== *

42 40 66:============== *

44 25 72:========= *

46 84 74:========================*===

48 140 71:=======================*=======================

50 86 65:=====================*=======

52 128 57:==================*========================

54 42 48:============== *

56 27 40:========= *

58 42 33:==========*===

60 8 27:=== *

62 7 22:=== *

64 8 17:=== *

66 3 14:= *

68 1 11:= *

70 22 8:==*=====

72 0 7: *

74 0 5: *

76 0 4: *

78 0 3:*

80 3 2:*

82 0 2:*

84 0 1:*

86 0 1:*

88 0 1:* inset = represents 1 library sequences

90 1 1:*

92 0 1:* :*

94 0 0: *

96 0 0: *

98 0 0: *

100 0 0: *

102 0 0: *

104 0 0: *

106 0 0: *

108 0 0: *

110 0 0: *

112 0 0: *

114 0 0: *

116 0 0: *

118 0 0: *

>120 0 0: *

15265 residues in 786 sequences

Statistics: MLE_cen statistics: Lambda= 0.2600; K=0.2484 (cen=39)

Kolmogorov-Smirnov statistic: 0.1102 (N=21) at 44

Algorithm: Smith-Waterman (SSE2, Michael Farrar 2006) (6.0 Mar 2007)

Parameters: BL50 matrix (15:-5), open/ext: -10/-2

Scan time: 0.000

The best scores are: s-w bits E(786)

[BaAMP:340](http://www.baamps.it/browse?task=peptide.display&ID=340)  pepti ( 12) 36 15.5 2.4

[BaAMP:217](http://www.baamps.it/browse?task=peptide.display&ID=217)  pepti ( 17) 32 14.0 9.7

[BaAMP:217](http://www.baamps.it/browse?task=peptide.display&ID=217)  pepti ( 17) 32 14.0 9.7

[BaAMP:218](http://www.baamps.it/browse?task=peptide.display&ID=218)  pepti ( 17) 32 14.0 9.7

>>[BaAMP:340](http://www.baamps.it/browse?task=peptide.display&ID=340)  peptide, (12 aa)

s-w opt: 36 Z-score: 90.6 bits: 15.5 E(): 2.4

Smith-Waterman score: 36; 83.3% identity (100.0% similar) in 6 aa overlap (5-10:7-12)

10

Pep19 RKKHVIRVCQDG

:::::.

#BIOFI RLCRIVVIRVCR

10

>>[BaAMP:217](http://www.baamps.it/browse?task=peptide.display&ID=217)  peptide, (17 aa)

s-w opt: 32 Z-score: 79.8 bits: 14.0 E(): 9.7

Smith-Waterman score: 32; 50.0% identity (60.0% similar) in 10 aa overlap (3-12:1-10)

10

Pep19 RKKHVIRVCQDG

: .::: :

#BIOFI KWCFRVCYRGICYRKCR

10

>>[BaAMP:217](http://www.baamps.it/browse?task=peptide.display&ID=217)  peptide, (17 aa)

s-w opt: 32 Z-score: 79.8 bits: 14.0 E(): 9.7

Smith-Waterman score: 32; 50.0% identity (60.0% similar) in 10 aa overlap (3-12:1-10)

10

Pep19 RKKHVIRVCQDG

: .::: :

#BIOFI KWCFRVCYRGICYRKCR

10

>>[BaAMP:218](http://www.baamps.it/browse?task=peptide.display&ID=218)  peptide, (17 aa)

s-w opt: 32 Z-score: 79.8 bits: 14.0 E(): 9.7

Smith-Waterman score: 32; 50.0% identity (60.0% similar) in 10 aa overlap (3-12:1-10)

10

Pep19 RKKHVIRVCQDG

: .::: :

#BIOFI KWCFRVCYRGICYRKCR

10

20>>>Pep20 - 12 aa

Library: BIOFIN/ssearch-baamp/biofin 15265 residues in 786 sequences

opt E()

< 20 0 0:

22 0 0: one = represents 2 library sequences

24 0 0:

26 0 0:

28 0 0:

30 0 1:*

32 0 4: *

34 24 11:=====*======

36 39 23:===========*========

38 36 39:================== *

40 56 54:==========================*=

42 51 66:========================== *

44 95 72:===================================*============

46 80 74:====================================*===

48 39 71:==================== *

50 58 65:============================= *

52 76 57:============================*=========

54 37 48:=================== *

56 11 40:====== *

58 13 33:======= *

60 45 27:=============*=========

62 21 22:==========*

64 60 17:========*=====================

66 14 14:======*

68 23 11:=====*======

70 3 8:== *

72 3 7:== *

74 2 5:= *

76 0 4: *

78 0 3: *

80 0 2:*

82 0 2:*

84 0 1:*

86 0 1:*

88 0 1:*

90 0 1:*

92 0 1:*

94 0 0:

96 0 0:

98 0 0:

100 0 0:

102 0 0:

104 0 0:

106 0 0:

108 0 0:

110 0 0:

112 0 0:

114 0 0:

116 0 0:

118 0 0:

>120 0 0:

15265 residues in 786 sequences

Statistics: MLE_cen statistics: Lambda= 0.1931; K=0.1076 (cen=39)

Kolmogorov-Smirnov statistic: 0.0553 (N=21) at 58

Algorithm: Smith-Waterman (SSE2, Michael Farrar 2006) (6.0 Mar 2007)

Parameters: BL50 matrix (15:-5), open/ext: -10/-2

Scan time: 0.010

!! No sequences with E() < 10.000000

240 residues in 20 query sequences

15265 residues in 786 library sequences

Scomplib [35.04]

start: Sat Feb 27 18:02:36 2016 done: Sat Feb 27 18:02:36 2016

Total Scan time: 0.020 Total Display time: 0.030

Function used was SSEARCH [version 35.04 Jul. 20, 2008]
